# A Gigantic Sarcopterygian (Tetrapodomorph Lobe-Finned Fish) from the Upper Devonian of Gondwana (Eden, New South Wales, Australia)

**DOI:** 10.1371/journal.pone.0053871

**Published:** 2013-03-06

**Authors:** Ben Young, Robert L. Dunstone, Timothy J. Senden, Gavin C. Young

**Affiliations:** 1 Lithicon Australia Pty Ltd, Canberra, Australian Capital Territory, Australia; 2 Research School of Earth Sciences, Australian National University, Canberra, Australian Capital Territory, Australia; 3 Research School of Physics & Engineering, Australian National University, Canberra, Australian Capital Territory, Australia; Ludwig-Maximilians-Universität München, Germany

## Abstract

*Edenopteron keithcrooki* gen. et sp. nov. is described from the Famennian Worange Point Formation; the holotype is amongst the largest tristichopterids and sarcopterygians documented by semi-articulated remains from the Devonian Period. The new taxon has dentary fangs and premaxillary tusks, features assumed to be derived for large Northern Hemisphere tristichopterids (*Eusthenodon*, *Hyneria*, *Langlieria*). It resembles *Eusthenodon* in ornament, but is distinguished by longer proportions of the parietal compared to the post-parietal shield, and numerous differences in shape and proportions of other bones. Several characters (accessory vomers in the palate, submandibulars overlapping ventral jaw margin, scales ornamented with widely-spaced deep grooves) are recorded only in tristichopterids from East Gondwana (Australia-Antarctica). On this evidence *Edenopteron* gen. nov. is placed in an endemic Gondwanan subfamily Mandageriinae within the Tristichopteridae; it differs from the nominal genotype *Mandageria* in its larger size, less pointed skull, shape of the orbits and other skull characters. The hypothesis that tristichopterids evolved in Laurussia and later dispersed into Gondwana, and a derived subgroup of large Late Devonian genera dispersed from Gondwana, is inconsistent with the evidence of the new taxon. Using oldest fossil and most primitive clade criteria the most recent phylogeny resolves South China and Gondwana as areas of origin for all tetrapodomorphs. The immediate outgroup to tristichopterids remains unresolved – either *Spodichthys* from Greenland as recently proposed, or *Marsdenichthys* from Gondwana, earlier suggested to be the sister group to all tristichopterids. Both taxa combine two characters that do not co-occur in other tetrapodomorphs (extratemporal bone in the skull; non-cosmoid round scales with an internal boss). Recently both ‘primitive’ and ‘derived’ tristichopterids have been discovered in the late Middle Devonian of both hemispheres, implying extensive ghost lineages within the group. Resolving their phylogeny and biogeography will depend on a comprehensive new phylogenetic analysis.

## Introduction

Lobe-finned fishes (Sarcopterygii), represented only by the coelacanth *Latimeria* and three lungfish genera in the modern fish fauna, were much more diverse during the Devonian Period. At that time they were the major group of osteichthyans (bony fishes); in contrast, the ray-finned fishes (Actinopterygii), which dominate the aquatic environment today, were relatively insignificant. Two major subdivisions are recognized for Devonian sarcopterygians [1]: Tetrapodomorpha and Dipnomorpha. Amongst Devonian tetrapodomorphs the family Tristichopteridae has been studied in great detail because of an assumed close relationship to the first land vertebrates (tetrapods). The most typical and best studied tristichopterid is *Eusthenopteron foordi* from the Late Devonian (Frasnian) of Miguasha, Canada [2]–[5]. *Marsdenichthys* Long, 1985 [6] from rocks of similar age in Victoria, Australia, was described as a possible very primitive tristichopterid from the Southern Hemisphere (recently redescribed [7]), and *Notorhizodon* Young et al., 1992 [8] is a very large sarcopterygian from the Middle Devonian (Givetian [9]) Aztec Siltstone of southern Victoria Land, Antarctica (initially assigned to the family Rhizodontidae; later re-interpreted as a tristichopterid [10]).

Because of their phylogenetic placement within the tetrapodomorph fishes, as the immediate sister group to elpistostegid fishes plus tetrapods [11], the biogeography of tristichopterids has been used to support a Gondwanan origin for tetrapods [12]. Since then, the occurrence of tetrapod trackways in older strata in Australia and Poland [13], [14] has introduced much uncertainty regarding where and when the first tetrapods evolved.

Much new information on East Gondwana tristichopterids resulted from descriptions of *Mandageria* Johanson and Ahlberg, 1997 [15] and *Cabonnichthys* Ahlberg and Johanson, 1997 [16], based on articulated material from the Frasnian (Late Devonian) Canowindra locality of central New South Wales (NSW). In addition, isolated skull and jaw bones from the Grenfell fossil fish assemblage of central NSW (Hunter Siltstone; Famennian), were referred to *Eusthenodon* [17], [18], another large Northern Hemisphere tristichopterid first described [19] in association with the tetrapods *Ichthyostega* and *Acanthostega* from the latest Devonian (Famennian) of East Greenland. *Mandageria* from Canowindra was first interpreted to be more closely related to *Eusthenodon* than to the associated *Cabonnichthys* [11], [16], but alternatively Young [20] noted characters indicating that the two Canowindra genera should belong in their own subfamily, including extra paired dermal bones in the palate (‘accessory vomers’) in both Canowindra genera.

Significantly, we have now identified these accessory vomers in the new taxon described below, thus demonstrating this character in at least three genera, from three separate localities, and at least two different ages, within the Late Devonian. All of these occurrences are located in southeastern Australia. Although homologous or analogous bones occur in Devonian ray-finned fishes [15], [16], the accessory vomers are unknown in any Northern Hemisphere Devonian lobe-fin, even though there are over 70 named genera of non-dipnoan sarcopterygians.

A Laurussian origin for tristichopterids was proposed [16] because presumed basal tristichopterids (*Tristichopterus*, *Eusthenopteron*, *Jarvikina*, *Platycephalichthys*) are all Northern Hemisphere forms. A later expansion into Gondwana, and a possible Gondwanan origin for derived tristichopterids, was also suggested [10].

**Figure 1:**
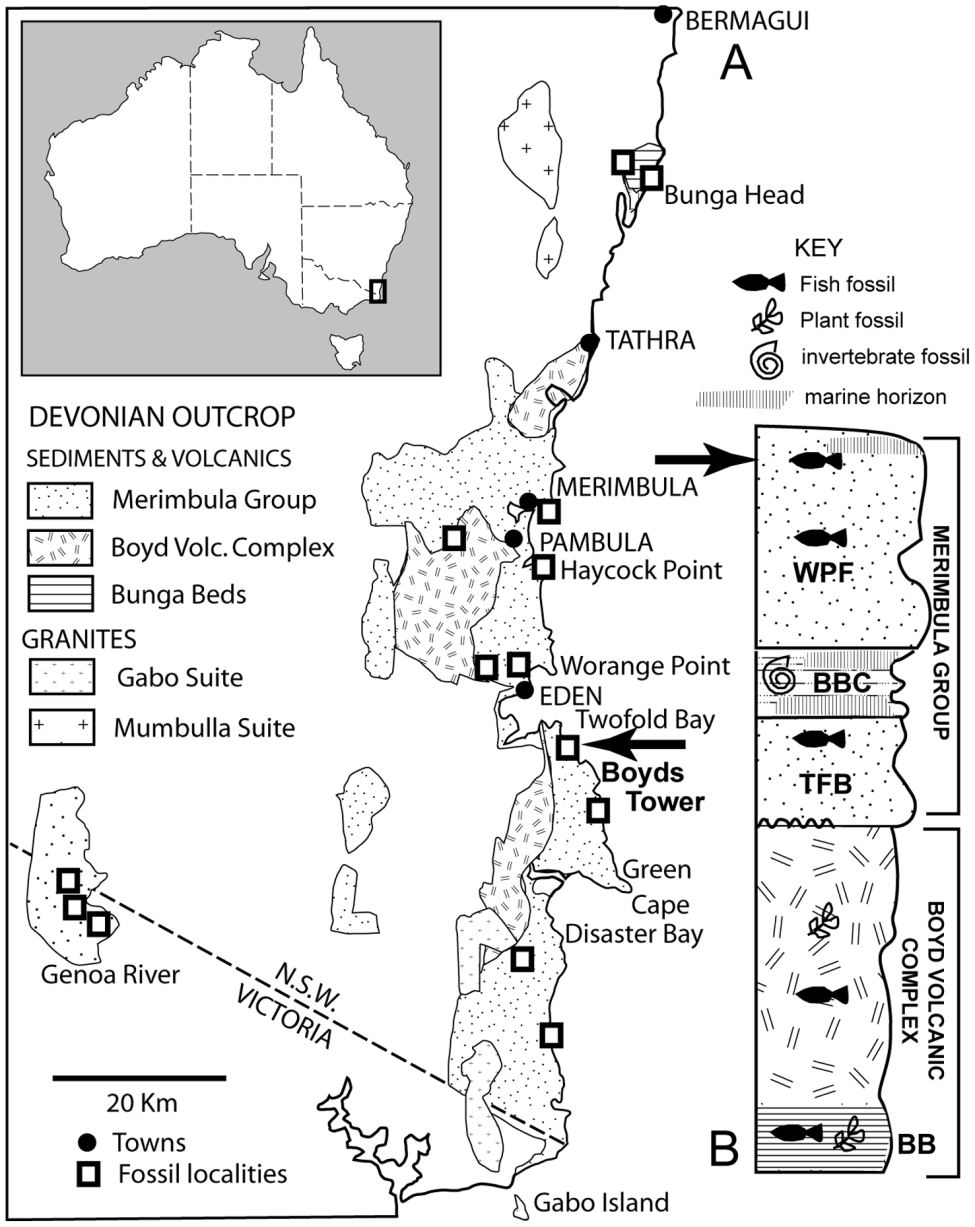
Locality details for *Edenopteron keithcrooki* gen. et sp. nov. **A**, geological map, and **B**, stratigraphic section showing (black arrows) the type locality (Boyds Tower) and horizon for *Edenopteron keithcrooki* gen. et sp. nov. **Abbreviations** (stratigraphic units): **BB**, Bunga Beds; **TFB**, Twofold Bay Formation; **BBC**, Bellbird Creek Formation; **WPF**, Worange Point Formation. For more detail see [22].

Another occurrence of the derived tristichopterid ‘*Eusthenodon*’ from the Famennian Worange Point Formation of south-eastern NSW south of Eden [21] represents the same sedimentary formation that has produced the new taxon *Edenopteron* gen. nov. described below. That material occurs at a site about 10 km down the coast from the type locality for *Edenopteron* gen. nov. (Boyds Tower, Fig. 1A), and some differences from *Eusthenodon* were noted based on a preliminary field assessment [20], but the material is either uncollected or unprepared (housed in the Australian Museum, Sydney), and was not considered further in this study. Both localities have similar red mudstone lithology, and presumably represent similar levels near the top of the Worange Point Formation (Fig. 1B), but correlation of different stratigraphic sections along these coastal exposures is difficult due to kink folding [22].

The fossil site near Boyds Tower producing *Edenopteron* was first discovered, and numerous samples with bone layers collected, by G. C. Young and R. W. Brown in 1979. These were treated with hydrochloric acid to remove the bone before latex rubber casting, but almost all specimens produced only fragmented bones of the placoderm *Remigolepis*. The single specimen of interest from the original collection was an internal impression of an articulated *Remigolepis* armor.

**Figure 2:**
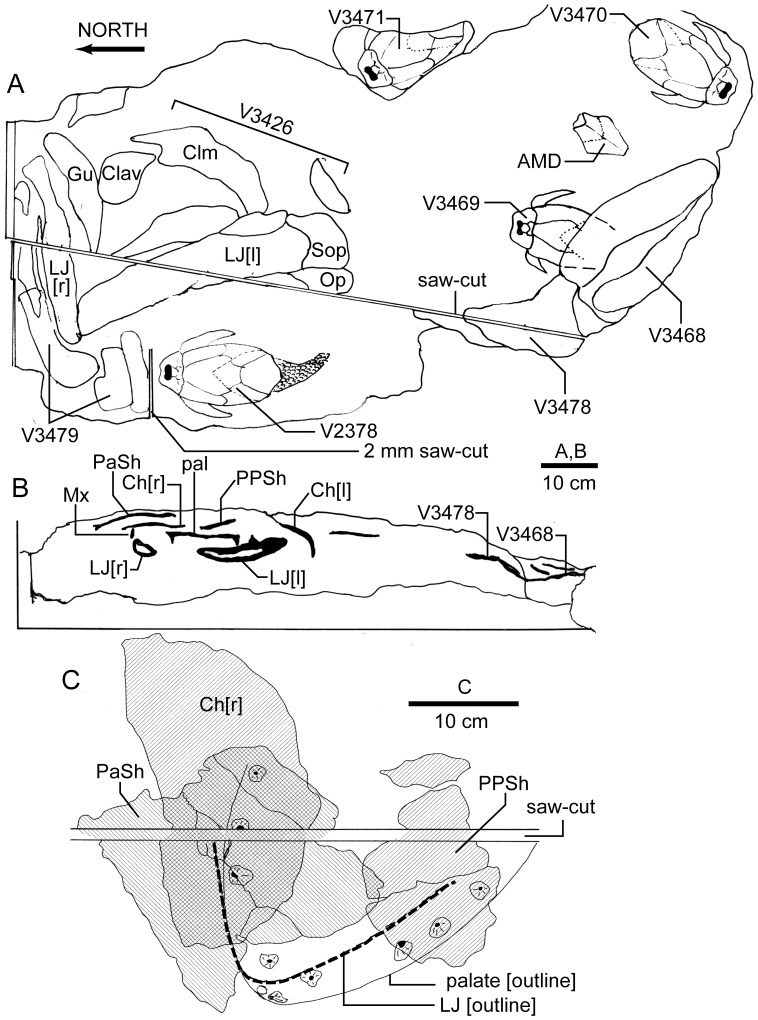
Excavation site for *Edenopteron keithcrooki* gen. et sp. nov. A , plan of site, showing original position of four sarcopterygians (ANU V3426, V3468, V3478, V3479) and four *Remigolepis* (ANU V2378, V3469, V3470, V3471), and the saw-cuts made to extract the first *Remigolepis* (ANU V2378); 2 mm saw-cut in the laboratory separated this from the *Edenopteron* holotype (ANU V3426), of which only the lowermost layer is shown. **B**, section of main saw-cut viewed from the west after removal of the block containing V2378 (*Remigolepis*). **C**, layout and layering of ANU V3426, showing the original position of the palate and lower jaw (LJ) as outlines, the middle layer (hatched, right slope) containing the displaced right cheek and post-parietal shield, and upper layer (hatched, left slope) containing the parietal shield.

In August 2006 the original fossil site was relocated, and the counterpart of this articulated *Remigolepis* armor was found by B. Young on a lichen-covered rock surface, representing a bed about 30 cm beneath the bone-bed layer. In early 2008 the block containing this armor was removed with a rock saw, on the corner of which a large vomerine fang was observed, with sections of a skull and jaws visible within the saw-cut (Fig. 2). Follow up excavations during April, October and December 2008 removed the entire specimen, the holotype of the new taxon described below, together with parts of several other sarcopterygians, and articulated *Remigolepis* armors.

## Materials and Methods

All necessary permits were obtained for the described study, which complied with all relevant regulations. Fieldwork in the Ben Boyd National Park was conducted under Scientific License S11982 issued by the NSW National Parks and Wildlife Service (NSW Office of Environment and Heritage). The original *Remigolepis* (ANU V2378) was excavated (16 January 2008) by means of two saw-cuts, approximately at right angles, and then broken free in two pieces using hammers and chisels to split a deeper bedding plane. The final excavation (by the four co-authors; 14–18 December 2008) involved removal of adjacent rock through a bedding thickness of 25–30 cm as one large block (∼116×80 cm) plus 150–200 associated pieces, extracted in sections by drilling over and under the specimen and splitting with chisels and wedges. Three additional *Remigolepis* specimens (ANU V3469, 3470, 3471), and remains of probably four sarcopterygians (ANU V3426, 3468, 3478, 3479) were recovered. However, most of these sarcopterygian remains belong to one specimen, representing the holotype of our new taxon (ANU V3426). During laboratory preparation an alphanumeric system was devised to number all pieces as they fit back together, the letter denoting the layer (e.g. a–d, the highest to lowest layers preserving the holotype), and adjacent pieces within each layer numbered sequentially as far as practicable. These labels are referred to in the descriptions. Numerous pieces were glued back together, and the final curated material comprises ∼80 separate pieces, the largest of which are 60×60 cm in size (see Information S1).

The layout of the block in situ (Fig. 2A) shows the relative position of specimens before extraction. The layering of the cut section, and relative position of the main components of the holotype (Fig. 2B–C) show it was preserved with both lower jaws meeting anteriorly at the symphysis, the left jaw rolled outwards so its inner surface faces upwards, and the right jaw in a more vertical position. The mandibular joint on the right side was still in articulation with the endocast of the adductor fossa preserved as a steinkern of red mudstone matrix. The dermal bones of the palate have been rotated clockwise ∼20° around an axis near the lower jaw symphysis, but with the teeth of the right maxilla and dentary still opposed and only slightly displaced, as are the anterior fangs of the vomer and dentary. Above this both moieties of the skull roof are slightly displaced and rotated further, the post-parietal shield in a clockwise direction, and the parietal shield back in an anti-clockwise direction. Our interpretation is that the decayed carcass, having been trapped in a dried-out billabong, was later flushed by a gentle current that lifted and rotated the skull roof and palate. The upper marginal dermal bones of the mouth were interlocked with the lower jaws, which remained immovably stuck in the mud. The parietal shield was then rotated anti-clockwise by another gentle current, coming to rest slightly above and overlapping the post-parietal shield (Fig. 2C). Similarly, one of the adjacent *Remigolepis* specimens (ANU V3470) has its anterior median dorsal plate displaced about 15 cm from the rest of the articulated armor (AMD, Fig. 2A), whereas the original *Remigolepis* (ANU V2378) includes a tail with scales in articulation, indicating the low energy of the currents. The fact that the dermal bones of the skull and palate of the *Edenopteron* holotype were displaced independently but remained intact suggests that the neurocranium was poorly ossified or completely cartilaginous in this fish, and had already decomposed before the skeleton was covered by sediment. The preservation of closely packed layers comprising only dermal bones is in contrast to other forms (e.g. *Notorhizodon*, *Mandageria*) where the parietal shield and parasphenoid remained firmly connected by the ethmosphenoid ossification of the neurocranium [8], [23]. Both cheeks in ANU V3426 have collapsed inwards and slid laterally, the right cheek preserved on a level slightly above the post-parietal shield, with the lower margin of the jugal and lachrymal bones displaced ∼70 mm laterally from the upper margin of the maxilla, which stayed with the lower jaw. Similarly, the premaxillae retained their position with respect to the palate, even though the central part of the parietal shield was rotated out of alignment. The skull bones are preserved tightly packed, with only 2–4 mm of matrix between some bone layers.

The right vomer of the holotype was retained as preserved bone stabilised with Mowital dissolved in ethanol, and with its fang was scanned using the ANU high resolution XCT scanner [24]. Poorly preserved bone from much of the remaining material was removed mechanically after being softened in ∼30% hydrochloric acid to reveal external and internal impressions. Bone was retained on counterparts where it showed structure, for example radiating growth pattern from bone ossification centers. Rubber latex casts were made from the rock impressions, and both casts and impressions were whitened with ammonium chloride to facilitate detailed study and photography. Most impressions include remnants of bone, presumably partly remineralized, because it remained too hard to remove even after several acid immersions. Many skeletal elements are represented on several adjoining pieces, and could not be permanently glued together because it would obscure closely associated bones (the morphology between the vomers and the snout involves reassembly of nine separate pieces). Thus, many of the illustrations are whitened latex casts taken from composites of several (up to ten) pieces of the specimen temporarily fitted back together. All interpretations are based on detailed study of both whitened latex casts, and the corresponding original bone or impressions preserved in the rock matrix.

Skull reconstructions were based on digital images of a life size 3D model. Outlines of all bones were first transferred to 0.7 mm thick aluminium sheet, cut out, and bent into shape. Due to compaction the bones have many fractures, but evidence of cross-sectional shape is still preserved, for example the dorsolateral angles of the parietal shield, and the ventrolateral angle on the cleithrum. The aluminium cutouts were fitted to a styrofoam core made of glued vertical layers. Initially, the flattened skull of a crocodile-like shallow water predator was envisaged, but neither the shoulder girdle nor the cheek units would fit this profile. Layers were added and the styrofoam sanded back until a reasonable fit was obtained, on a profile approaching more that of *Eusthenopteron* as preserved at Miguasha ([5]: fig. 2B).

### Institutional Abbreviations

**AFM**, Age of Fishes Museum, Canowindra; **AMF**, Australian Museum, Sydney; **ANSP**, Academy of Natural Sciences, Philadelphia; **ANU V**, Australian National University, Canberra; **NMV**, Museum Victoria, Melbourne; **P**, Natural History Museum of Denmark, Copenhagen.

### Anatomical Abbreviations

**ac.Vo**, accessory vomer; **a.LJ**, anterior edge of subopercular abutting lower jaw; **asc.pr**, ascending process of parasphenoid; **b.a**, possible bone of attachment; **Ch(l, r)**, cheek unit (left, right); **Clav**, clavicle; **Clm**, cleithrum; **De**, dentary; **dent**, denticulate surface; **Dpl**, dermopalatine; **Ect**, ectopterygoid; **Ent**, entopterygoid; **Ent.tp**, entopterygoid toothplate; **e.pc**, extensions of pulp cavity between folds in the dentine; **f.bhp**, buccohypophyseal foramen; **f.Co**, coronoid fang; **f.Co_1_**, first coronoid fang; **f.De**, dentary fang; **f.Dpl**, dermopalatine fang; **f.Ect**, ectopterygoid fang; **fe.exa**, external nasal opening; **f.Ent**, entopterygoid fang; **fl**, possible flange enclosing anterior edge of cleithrum; **fo.hyp**, hypophyseal fossa; **fr**, possible fin ray; **f.Vo**, position of vomerine fang; **gr**, groove; **Gu**, gular; **Id_1–4_**, infradentaries 1–4; **IT**, intertemporal; **Ju**, jugal; **La**, lachrymal; **la.pal**, palatine lamina; **la.Vo**, tooth-bearing lamina of vomer; **LJ(l, r)**, lower jaw (left, right); **llc**, main lateral line sensory canal (or ridge enclosing it); **m.dent**, marginal dentition; **Mx**, maxilla; **n**, notch; **Na**, nasal; **nn**, nasal notch; **od.Clav**, overlap for clavicle; **od.Esc**, overlap for extrascapular; **od.IT**, overlap for intertemporal; **od.Ju**, overlap for jugal; **od.La**, overlap for lachrymal; **od.Pa/IT**, overlap on postorbital for parietal/intertemporal bones of skull; **od.Po**, overlap for postorbital; **od.Pop**, overlap for preopercular; **od.Pos**, overlap for postspiracular; **od.Qj**, overlap for quadratojugal; **od.R.l**, overlap for lateral rostral; **odSbm**, overlap on lower jaw for submandibulars; **od.So2**, overlap for posterior supraorbital; **od.Vo**, overlap for vomer; **Op**, opercular; **orb**, orbit; **orb.m**, orbital margin; **ost**, osteodentine; **Pa**, parietal; **pa**, posterior angle on clavicle margin; **PaSh**, parietal shield of skull roof; **pbl**, postbranchial lamina of cleithrum; **pc**, pulp cavity; **Pi**, pineal plate/s; **pi**, pineal opening; **p.ioc**, surface pits of infraorbital sensory canal; **pl**, pitline; **plic**, plicidentine; **pl.Id_2,4_**, pitline on infradentaries; **Pmx**, premaxilla; **Po**, postorbital; **Pop**, preopercular; **PPa**, postparietal; **ppr**, posterior process of vomer; **PPSh**, post-parietal shield of skull roof; **pr**, process; **pr.dim**, dermintermedius process; **pr.Mx**, maxillary process; **pr.psp**, post-spiracular process; **pr.te**, tectal process; **p.soc**, surface pits of supraorbital sensory canal; **Psp**, parasphenoid; **Ptra**, anterior median postrostral; **Ptrp**, posterior median postrostral; **Qj**, quadratojugal; **qj.ri**, ridge on inner surface of quadratojugal; **ri**, ridge; **riVo**, ridge inside anterior margin of vomer; **R.l**, lateral rostral; **Sbm**, submandibulars; **Sbm_1–4_**, posterior to anterior submandibulars; **sc**, scale; **Sclm**, supracleithrum; **Sh.g**, incomplete shoulder-girdle; **sm**, smooth zone on bone margin; **So1**, anterior supraorbital; **So2**, posterior supraorbital; **Sop**, subopercular; **spir**, spiracular notch/opening; **Sq**, squamosal; **St**, supratemporal; **Ta**, tabular; **Te**, tectal; **th**, thickening; **t.Pmx**, premaxillary tusk; **Vo**, vomer.

### Nomenclatural Acts

The electronic edition of this article conforms to the requirements of the amended International Code of Zoological Nomenclature, and hence the new names contained herein are available under that Code from the electronic edition of this article. This published work and the nomenclatural acts it contains have been registered in ZooBank, the online registration system for the ICZN. The ZooBank LSIDs (Life Science Identifiers) can be resolved and the associated information viewed through any standard web browser by appending the LSID to the prefix “http://zoobank.org/”. The LSIDs in ZooBank are as follows:

For this publication: urn:lsid:zoobank.org:pub:5B74E736-1489-4C86-AEAD-E0E47D5EC12D

For the new genus Edenopteron Young, Dunstone, Senden & Young, established within this publication: urn:lsid:zoobank.org:act:CD3B965B-CE7E-431E-9F05-13F5855455F5

For the new species Edenopteron keithcrooki Young, Dunstone, Senden & Young, established within this publication: urn:lsid:zoobank.org:act:F1223F27-87CE-4B32-B677-747C040A69FE.

The electronic edition of this work was published in a journal with an ISSN, and has been archived and is available from the digital repository PubMed Central, LOCKSS.

## Systematic Paleontology

Osteichthyes Huxley, 1880 [25].

Sarcopterygii Romer, 1955 [26].

Tetrapodomorpha Ahlberg, 1991 [1].

Tristichopteridae Cope, 1889 [27].

### Remarks

Recent papers [28]–[30] recognize only one unique character of the family Tristichopteridae (also known as ‘Eusthenopteridae’), the absence of the extratemporal and presence instead of a ‘post-spiracular’ bone in the skull (probably the same bone displaced posteriorly). Other features suggested to characterise tristichopterids, like the three-lobed caudal fin, and round scales that lack cosmine and have a median ridge on the inner surface, occur outside the group and may be primitive [15]. Within tristichopterids, two general morphological ‘models’ were summarized [10]: i) generally smaller forms like *Eusthenopteron* which lack anterior fangs on the dentary, tend to be stratigraphically older (Middle-Late Devonian), and are assumed to be phylogenetically basal within the group, and ii) larger (2–3 m long) presumably derived tristichopterids with dentary fangs in the jaws, which are mainly known from the Late Devonian. The first described in the second group was *Eusthenodon* Jarvik, 1952 [19] from the latest Devonian (Famennian) of East Greenland.

The new taxon described below also belongs in the latter group, together with other large tristichopterids presumed to be derived, such as *Platycephalichthys* Vorobyeva, 1959 [32], *Hyneria* Thomson, 1968 [33], *Notorhizodon* [8], *Mandageria* [15] and *Cabonnichthys* [16], the last three from the Southern Hemisphere (Australia and Antarctica). *Marsdenichthys* is another Australian taxon originally assessed as a primitive sister taxon to tristichopterids [6], and recent revision [7] retains the idea that it may lie outside the group, whereas Snitting [28] placed the Greenland taxon *Spodichthys* (which shares with *Marsdenichthys* a lateral extratemporal bone) as the sister group to tristichopterids.

In addition to the type locality of East Greenland, *Eusthenodon* sp. has been reported from Russia, Belgium, Pennsylvania, Australia, and possibly South Africa, but this widespread distribution needs support from detailed description. In Russia the species ‘*Eusthenodon’ wenjukovi* was placed in a new genus *Jarvikina* by Vorobyeva [34], who also erected a subfamily Platycephalichthyinae for *Platycephalichthys*. A ‘mandageriid’ grouping within some derived tristichopterids from Australia-Antarctica was suggested by Young [20], and the new genus and species described here conforms with the characters proposed to support that grouping. The most recent phylogenetic analyses of tristichopterids [28], [30] have not taken account of these new characters.

Mandageriinae Young, 2008 [20].

### Remarks

Two characters were proposed to support this familial/subfamilial grouping of tristichopterids, and both have been established in the new taxon described below: i) paired accessory vomers in the palate; ii) scales ornamented with deep subparallel grooves separated by broad and flat intervening ridges much wider than the grooves. Possible additional characters suggested by descriptions below include T-shaped supraorbital bones, quadrilateral lateral rostral bone, submandibular series overlapping infradentaries of the lower jaw rather than being overlapped by them, quadrilateral supracleithrum and triangular anocleithrum, and absence of basal scutes on fin lobes.

Edenopteron keithcrooki gen. et sp. nov.

Figs. 3, 4, 5, 6, 7, 8, 9, 10, 11, 12, 13, 14, 15, 16, 17, 18, 19A–B, 20, 21, 22, 23A–C.

**Figure 3:**
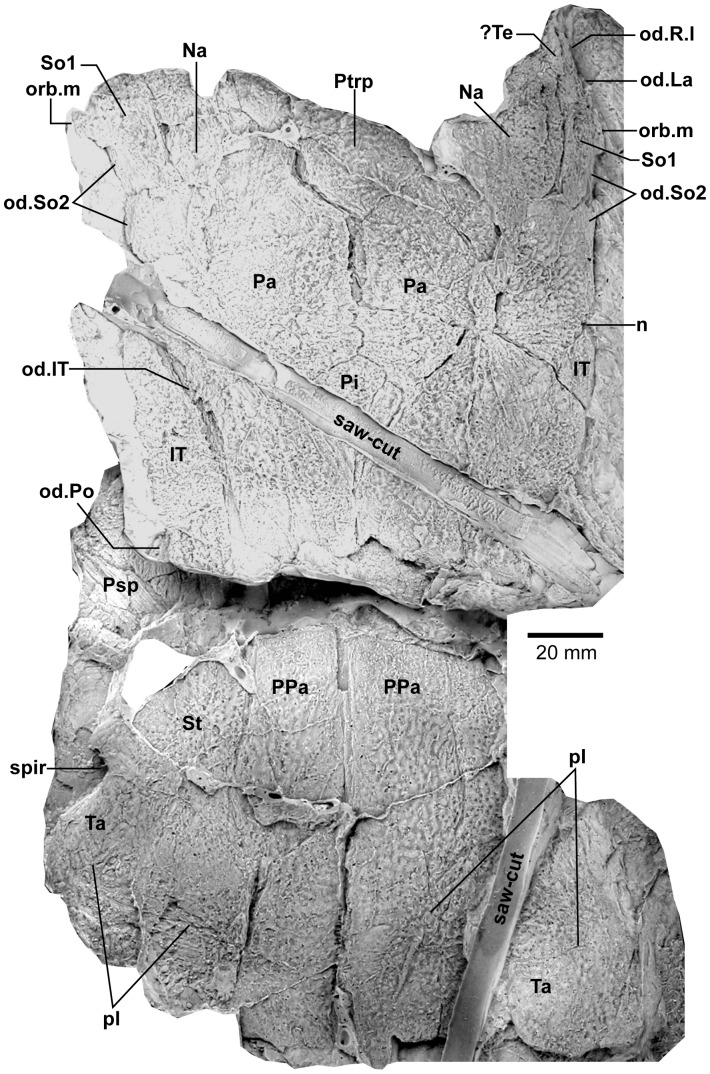
*Edenopteron keithcrooki* gen. et sp. nov. Holotype (ANU V3426). Parietal and post-parietal shields in approximate life position (latex casts whitened with ammonium chloride).

**Figure 4:**
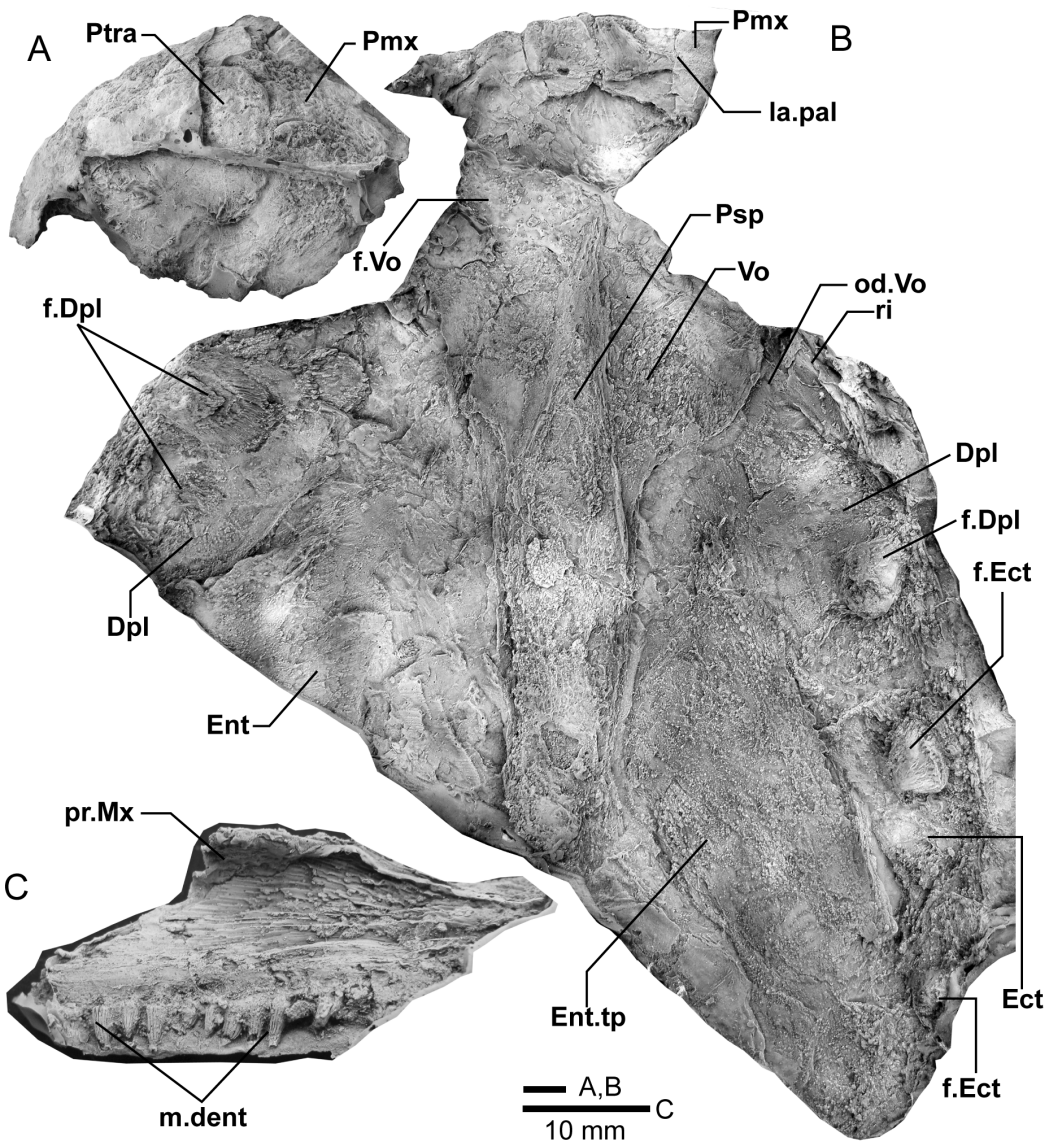
*Edenopteron keithcrooki* gen. et sp. nov. Holotype (ANU V3426). **A**, composite latex of pieces a7 (anterior) and a14 (posterior) showing snout in dorsal view; **B**, composite latex (a8, b7) showing internal snout surface, and ventral view of dermal bones of part of the palate. **C**, inner view of anterior end of right maxilla (latex casts whitened with ammonium chloride).

**Figure 5:**
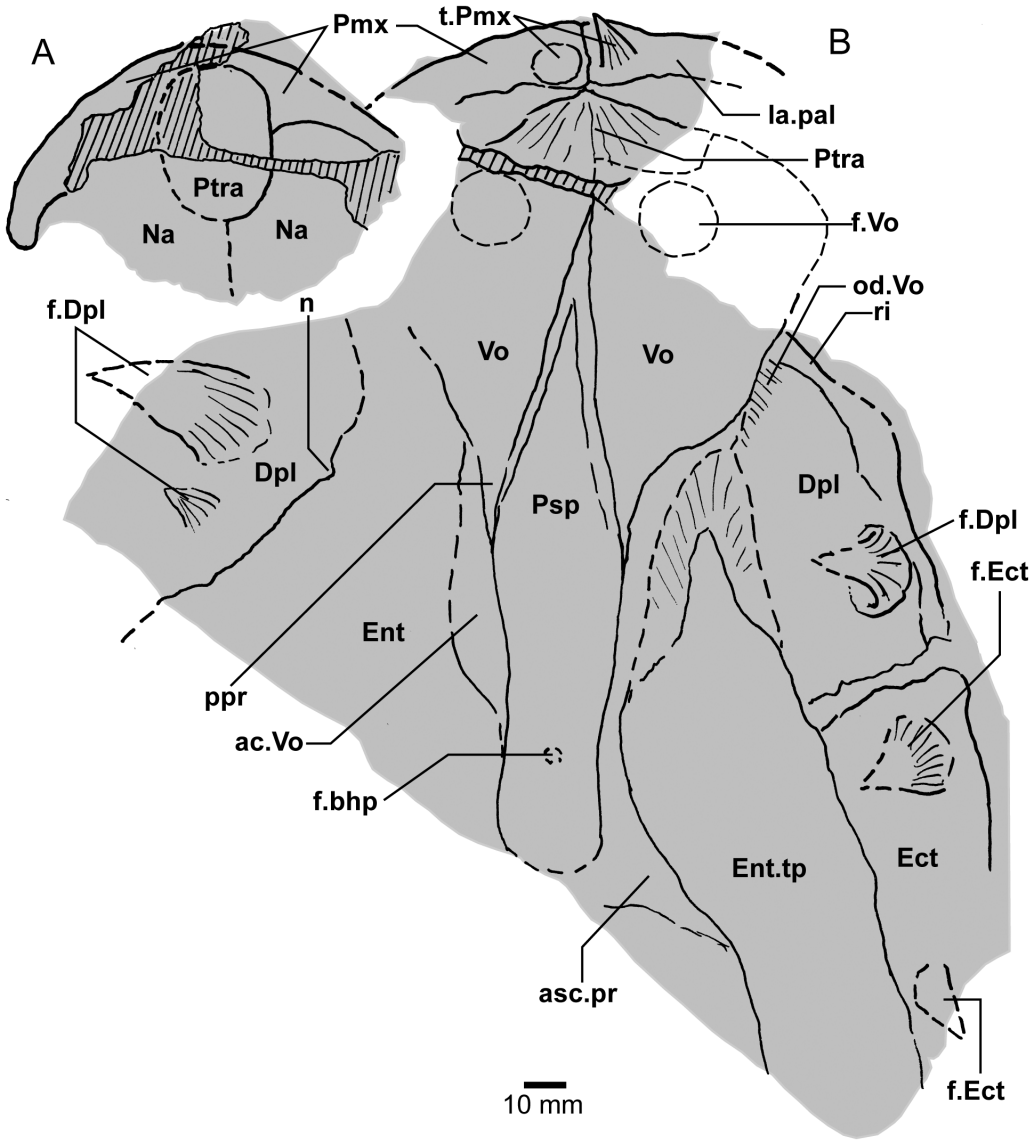
*Edenopteron keithcrooki* gen. et sp. nov. Holotype (ANU V3426). **A**, **B**, Interpretive outlines of bone sutures and other structures on the latex casts illustrated in Figure 4A, B.

**Figure 6:**
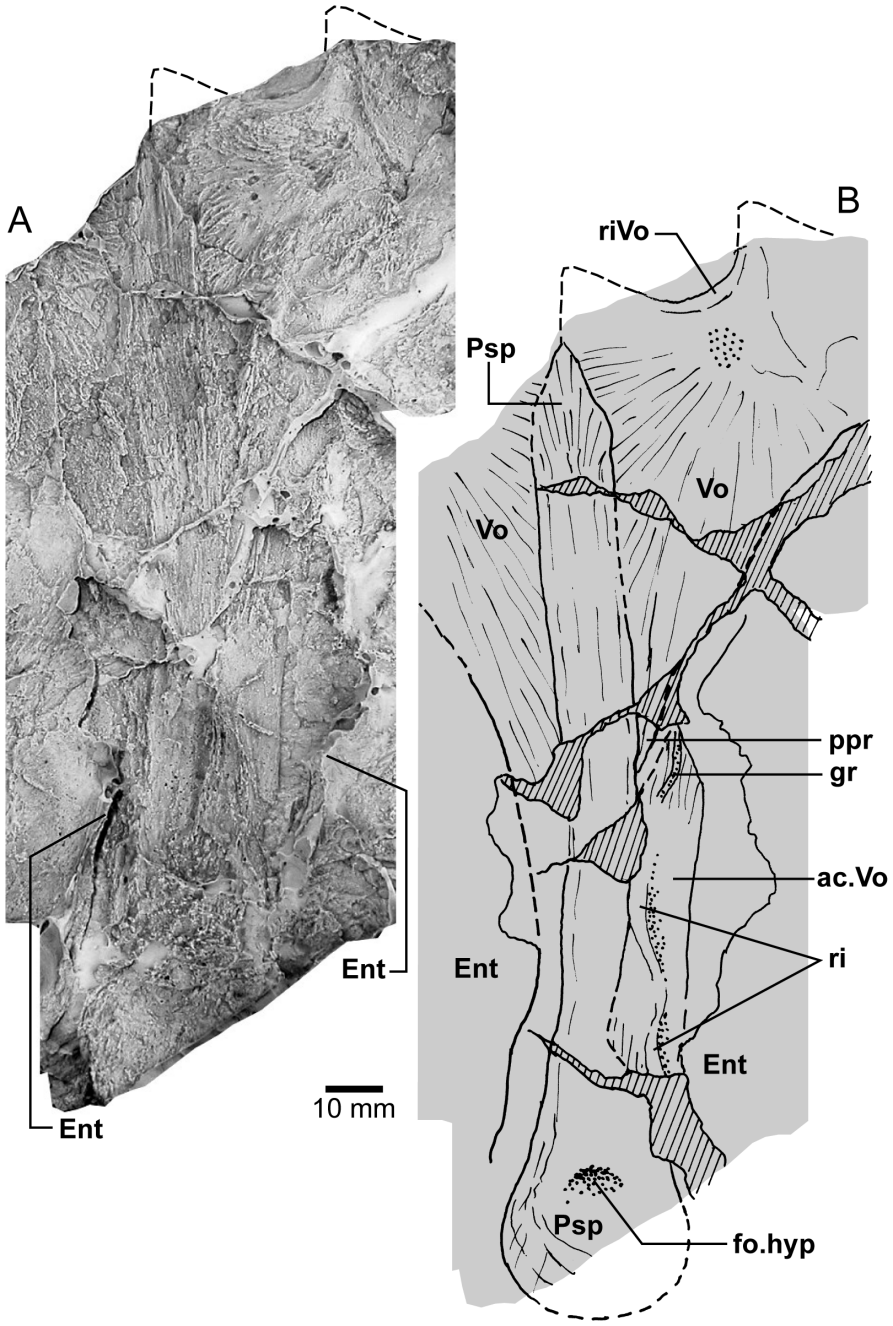
*Edenopteron keithcrooki* gen. et sp. nov. Holotype (ANU V3426). **A**, Composite latex showing the dorsal surface of some dermal bones of the palate (latex cast whitened with ammonium chloride). **B**, Interpretive outline of bone sutures and other structures of specimen in A. Anterior margin of vomer after piece a8 and Jarvik ([19]: fig. 29).

**Figure 7:**
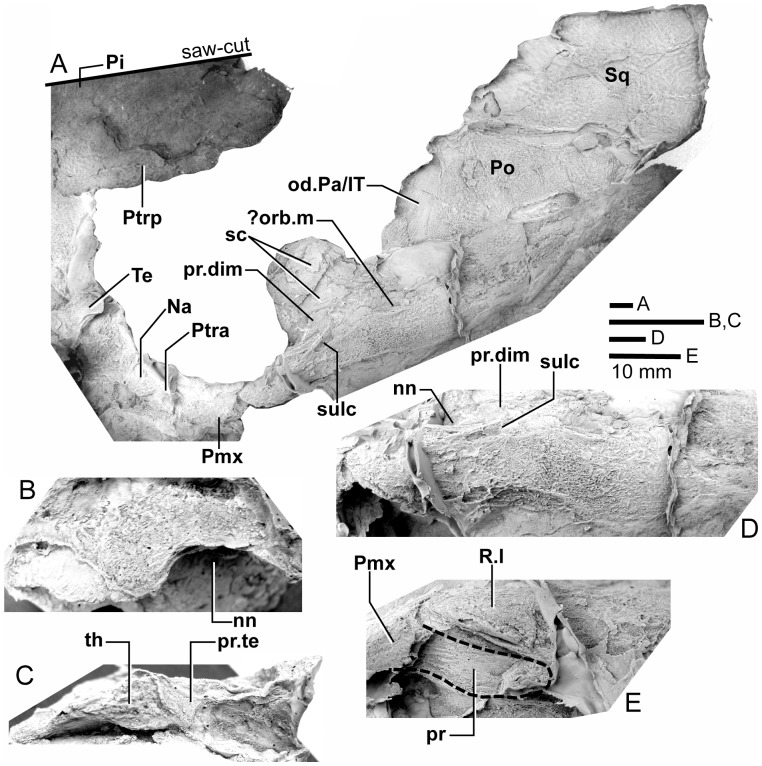
*Edenopteron keithcrooki* gen. et sp. nov. Holotype (ANU V3426). **A**, composite latex showing anterolateral view of part of the parietal shield (displaced) in relation to bones of the snout, left anterolateral margin of the skull and left cheek (anterior pointing downwards). **B**, presumed right tectal in external view, and ventral view (**C**). **D**, left lateral rostral bone in external view, and ventral view (**E**). (latex casts whitened with ammonium chloride).

**Figure 8:**
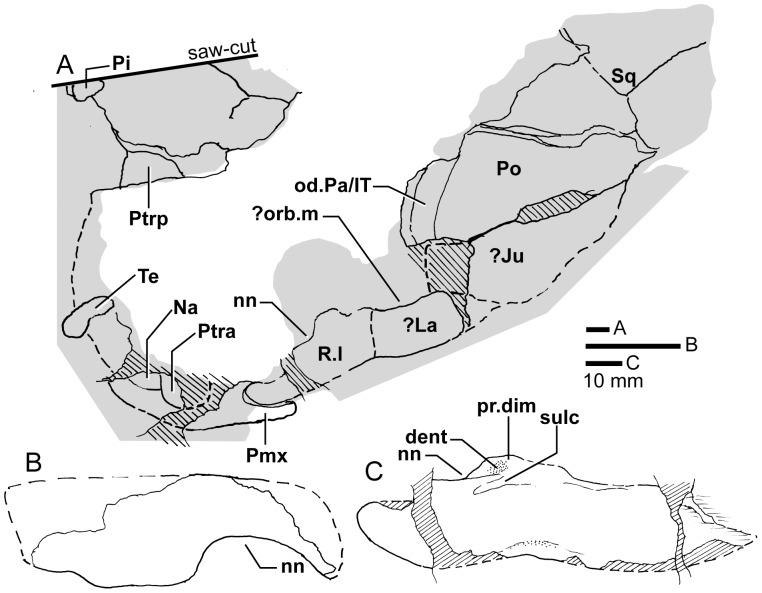
*Edenopteron keithcrooki* gen. et sp. nov. Holotype (ANU V3426). **A**, Interpretive outline of bone sutures and other structures of latex cast in Figure 7A. **B**, Interpretive outline of presumed right tectal shown in Figure 7B. **C**, Interpretive outline of left lateral rostral bone shown in Figure 7D.

**Figure 9:**
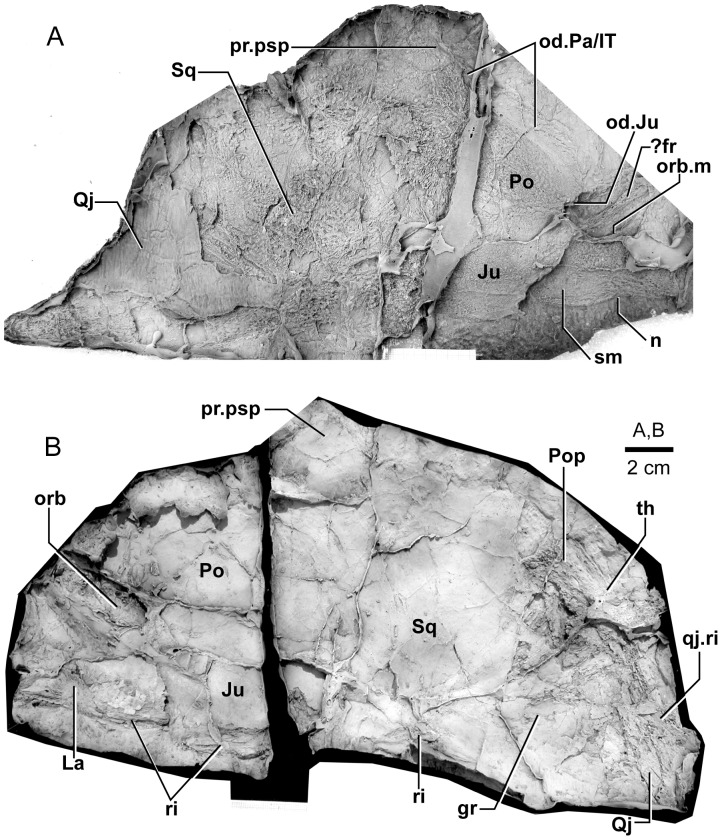
*Edenopteron keithcrooki* gen. et sp. nov. Right cheek unit of holotype (ANU V3426). **A**, external view; **B**, internal view (latex casts whitened with ammonium chloride).

**Figure 10:**
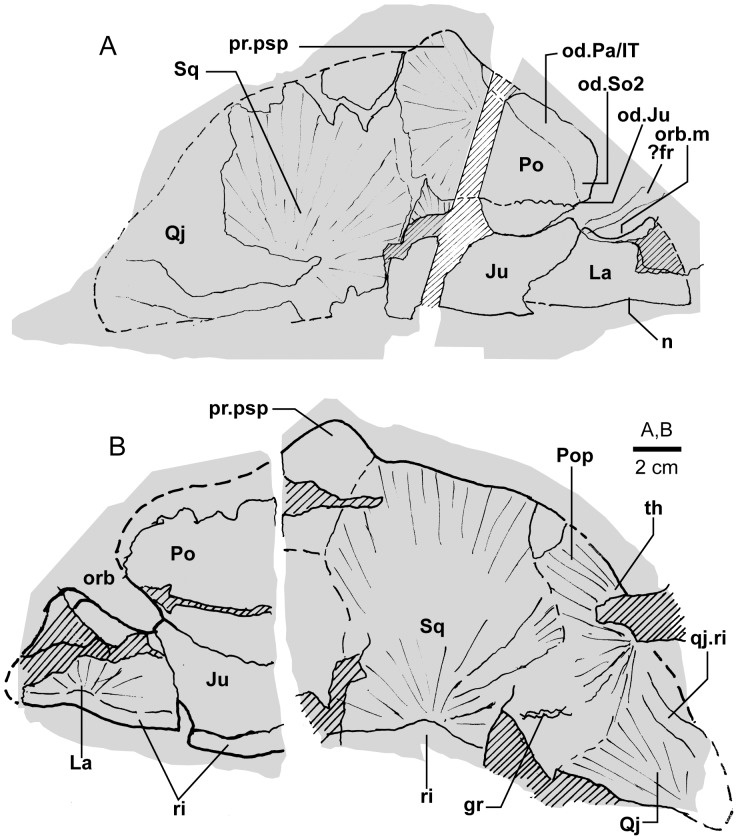
*Edenopteron keithcrooki* gen. et sp. nov. Right cheek unit of holotype (ANU V3426). **A**, **B**, Interpretive outlines of bone sutures and other structures shown in Figure 9A, B.

**Figure 11:**
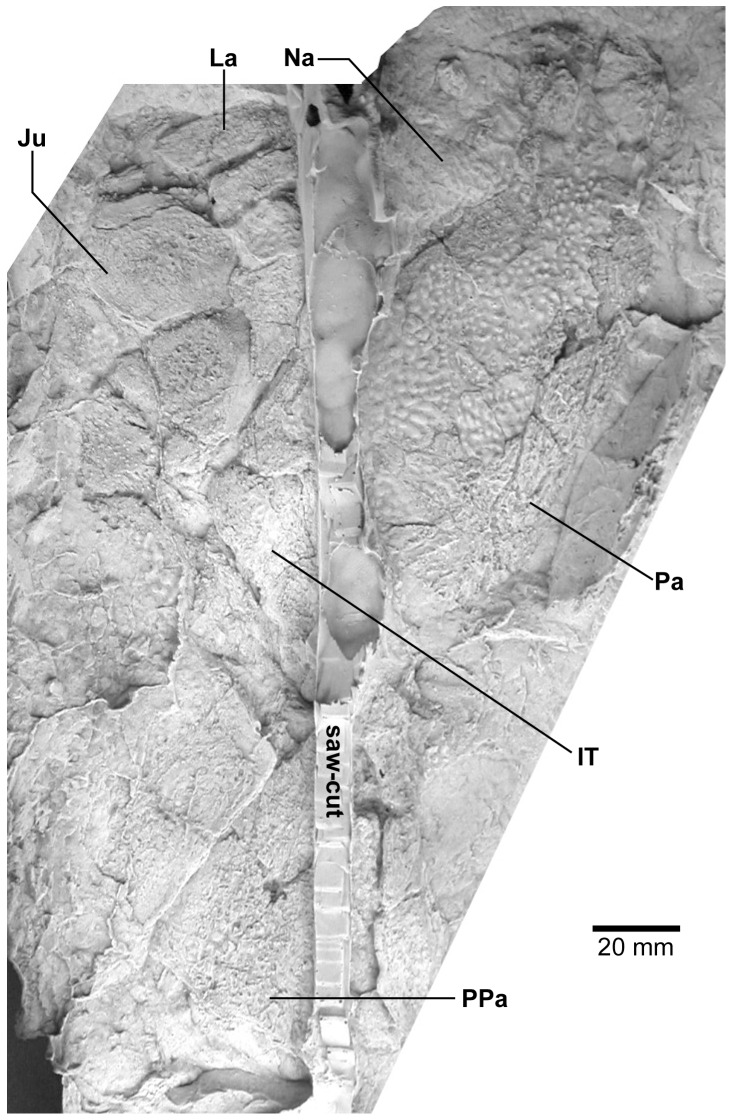
*Edenopteron keithcrooki* gen. et sp. nov. ANU V3478. Incomplete flattened skull and left cheek in dorsal view, preserved on pieces f4 (left side) and h1 (right side) (latex cast whitened with ammonium chloride).

**Figure 12:**
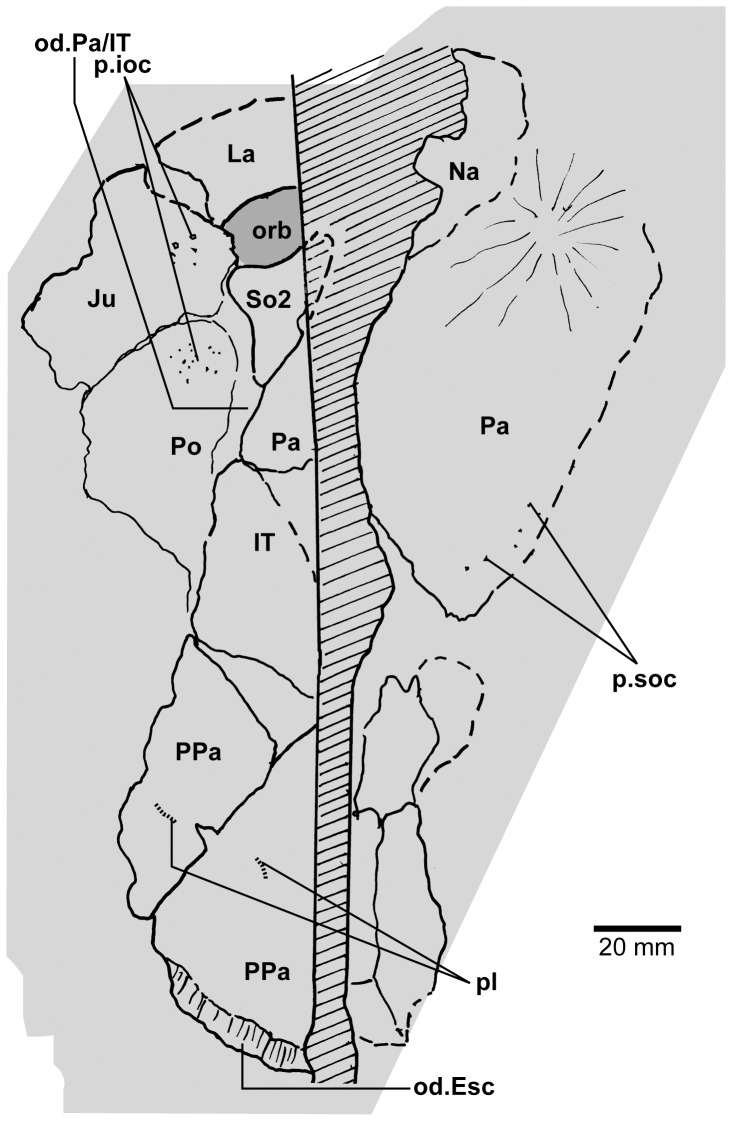
*Edenopteron keithcrooki* gen. et sp. nov. ANU V3478. Interpretive outline of bone sutures and other structures shown in Figure 11.

**Figure 13:**
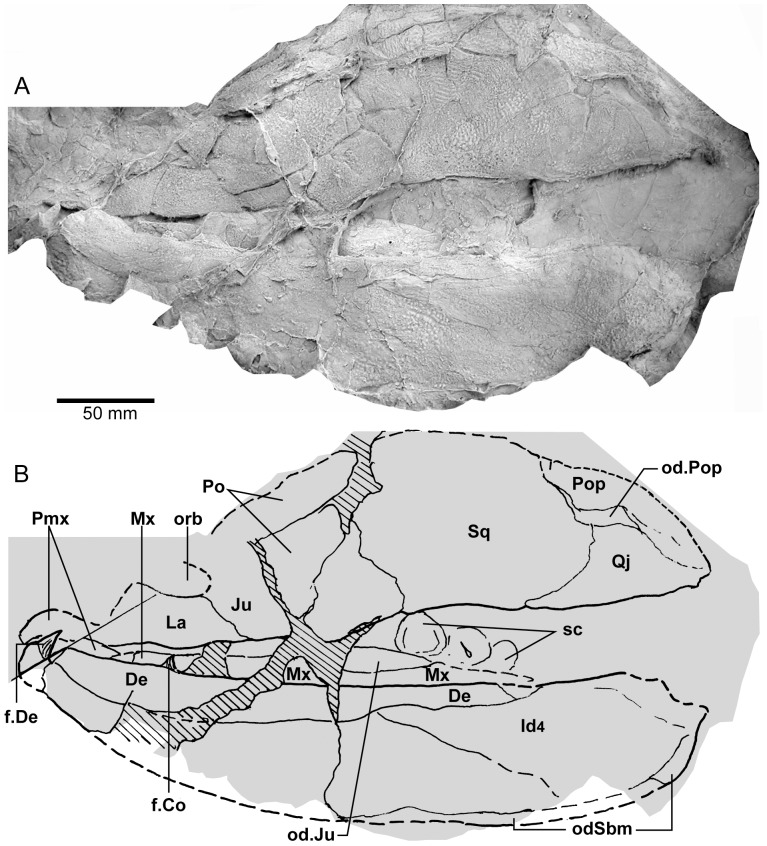
*Edenopteron keithcrooki* gen. et sp. nov. **A**, Paratype, ANU V3468, external view of left cheek and lower jaw (latex cast whitened with ammonium chloride). **B**, Interpretive outline of bone sutures and other structures shown in A.

**Figure 14:**
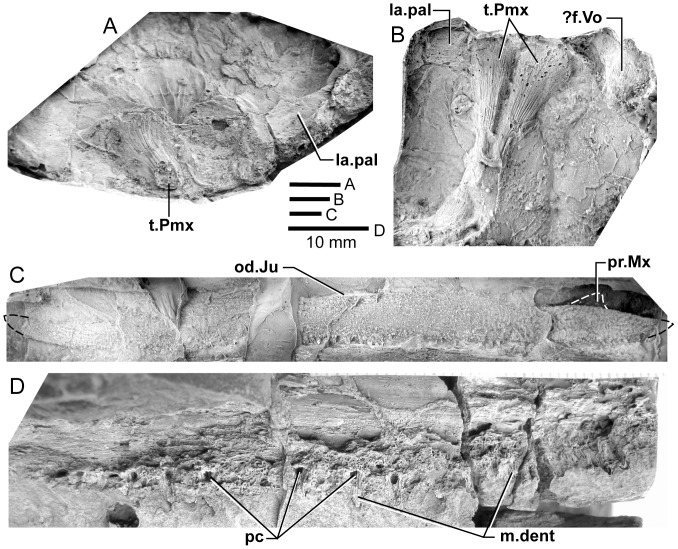
*Edenopteron keithcrooki* gen. et sp. nov. **A**, Holotype, ANU V3426, detail of the snout in internal view showing one premaxillary tusk and adjacent attachment surface (anterior pointing downwards; cf. Fig. 4B). **B**, ANU V3478, internal view of snout showing presumed premaxillary tusks compressed backwards over the premaxillae (anterior pointing upwards; extra fang on right side on either a displaced vomer or dermopalatine, but too incomplete to determine). **C**, Holotype (ANU V3426), composite latex of left maxilla in external view (for inner view of anterior end see Fig. 4C); **D**, Holotype (ANU V3426), internal view of posterior end of left maxilla (latex casts [A–C] and preserved bone [D] whitened with ammonium chloride).

**Figure 15:**
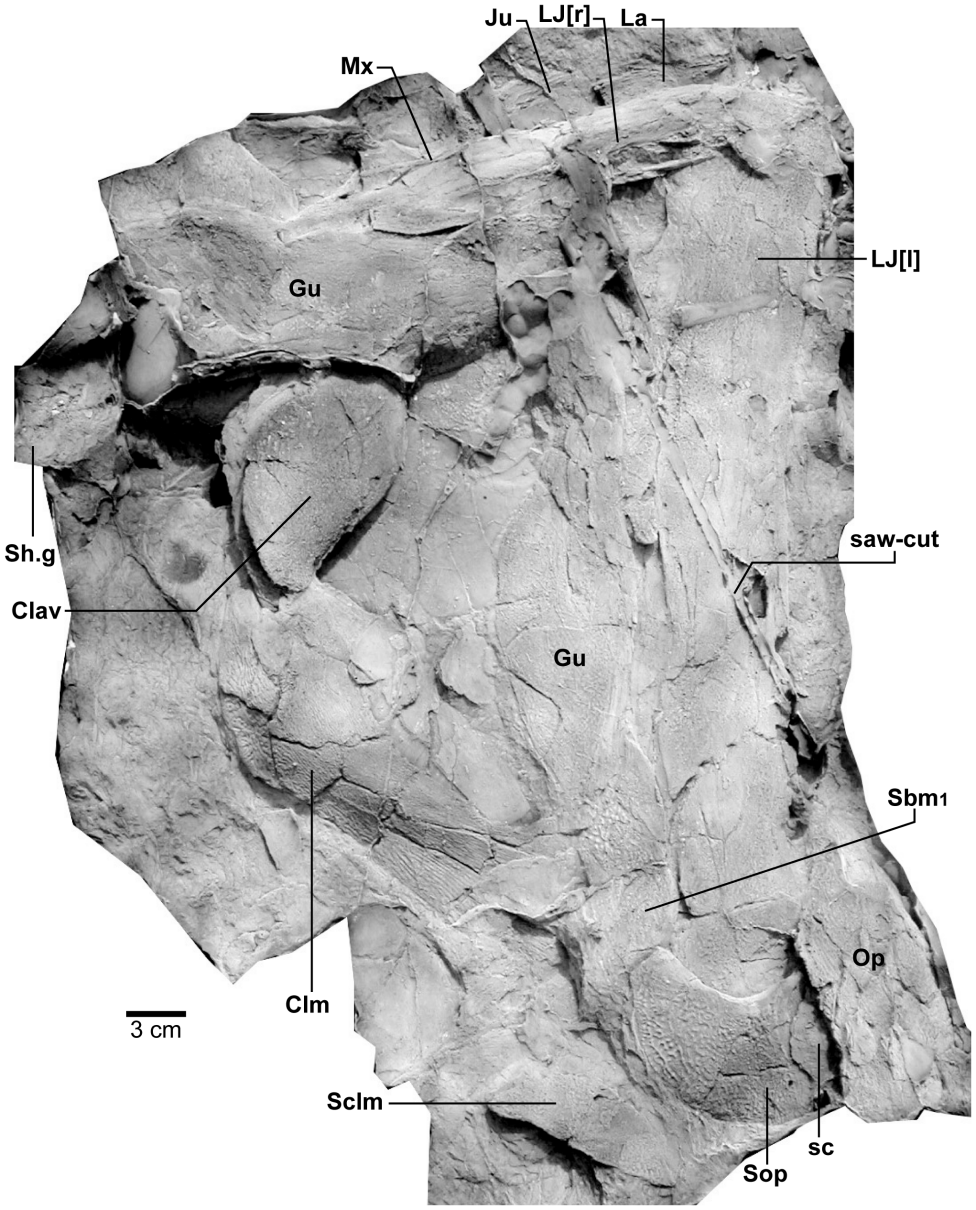
*Edenopteron keithcrooki* gen. et sp. nov. Holotype (ANU V3426). Composite latex showing both lower jaws, gulars, submandibulars, operculum and shoulder-girdle (latex cast whitened with ammonium chloride).

**Figure 16:**
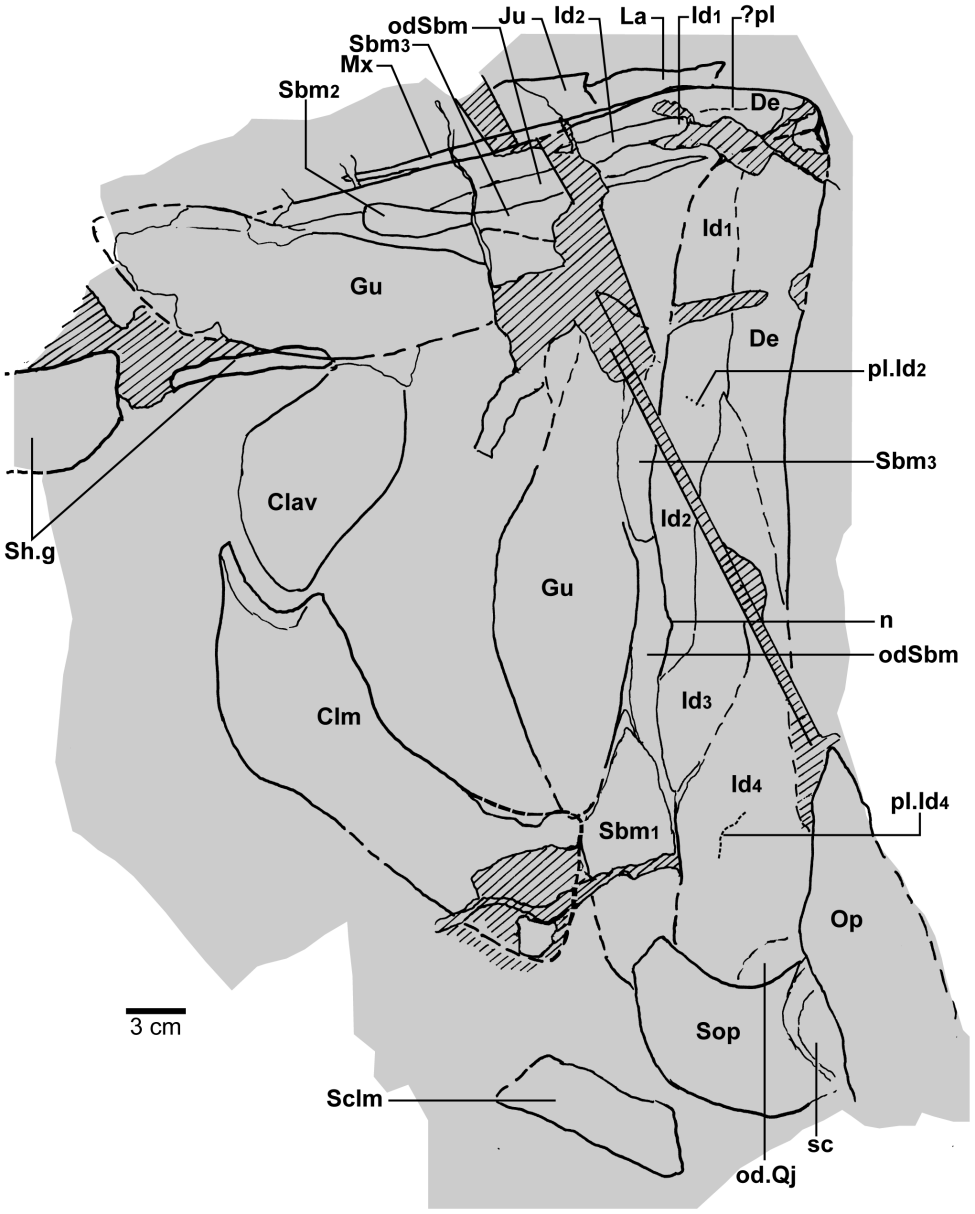
*Edenopteron keithcrooki* gen. et sp. nov. Holotype (ANU V3426). Interpretive outline of bone sutures and other structures on the composite latex of Figure 15.

**Figure 17:**
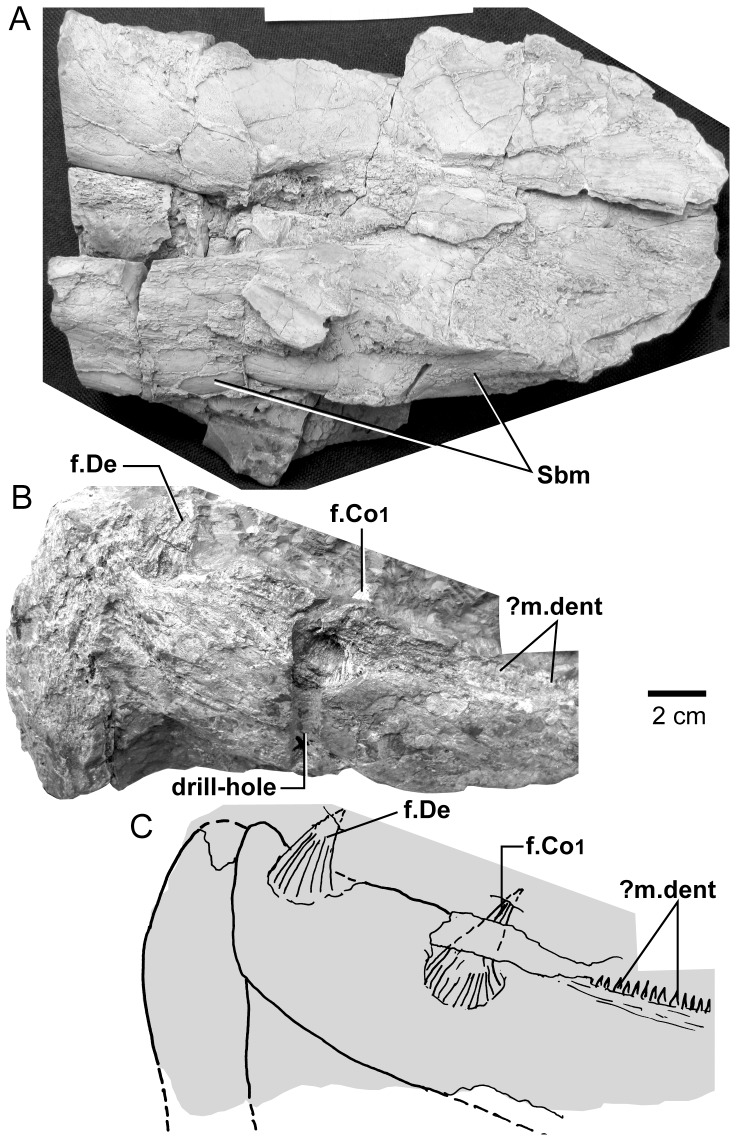
*Edenopteron keithcrooki* gen. et sp. nov. Holotype (ANU V3426). **A**, steinkern of left mandibular joint in lateral view (whitened with ammonium chloride). **B**, preserved bone of jaw symphysis in ventral view. **C**, Interpretive drawing of specimen in B.

**Figure 18:**
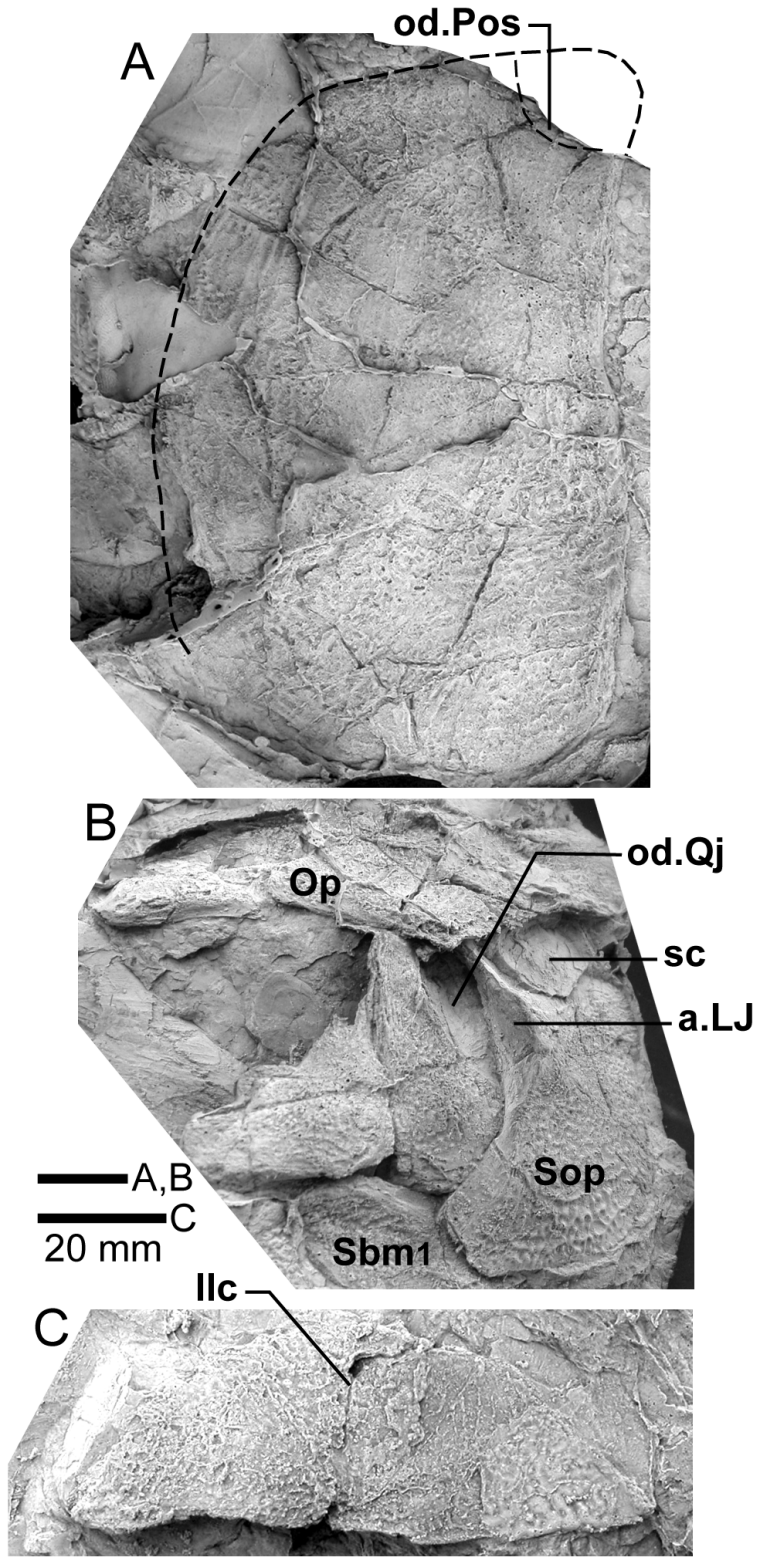
*Edenopteron keithcrooki* gen. et sp. nov. Holotype (ANU V3426). **A**, Right opercular bone, external view. **B**, left opercular and subopercular and adjacent bones, anterior view. **C**, presumed left supracleithrum, external view. (latex casts whitened with ammonium chloride).

**Figure 19:**
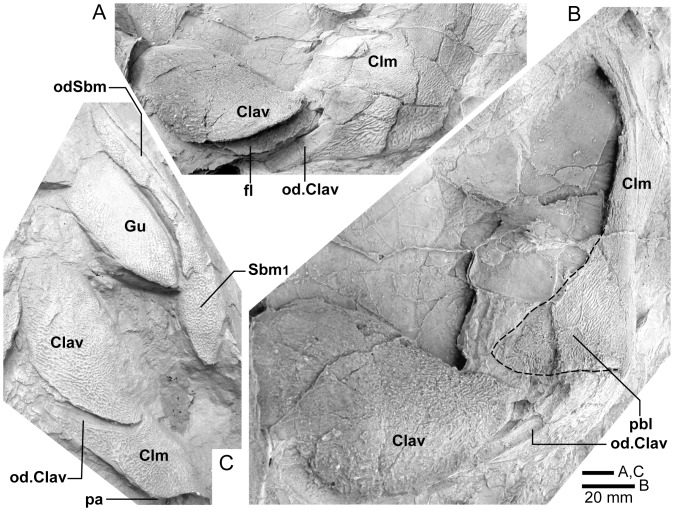
Comparison of shoulder-girdle bones. **A**, **B**, *Edenopteron keithcrooki* gen. et sp. nov. Holotype (ANU V3426), left cleithrum and clavicle (latex casts whitened with ammonium chloride). **A**, posteroventral view showing overlap surfaces; **B**, anterior view of cleithrum showing postbranchial lamina. **C**, *Mandageria fairfaxi* [15]. Cast from AFM ‘slab 191A’ showing the relationship between left cleithrum, clavicle, gular, submandibulars and lower jaw in ventral view (resin cast whitened with ammonium chloride).

**Figure 20:**
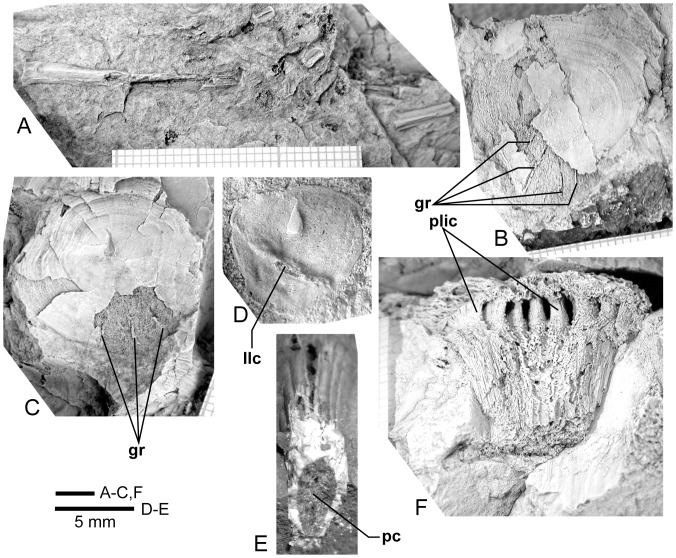
*Edenopteron keithcrooki* gen. et sp. nov. **A**, ANU V3478, piece f3 showing associated lepidotrichia presumed to come from the pectoral fin. **B**, internal view of a scale, partly broken to show impression of external surface, one example from a patch of at least five round scales 25–30 mm across preserved on piece g5 inside the cheek of ANU V3468. **C**, similarly preserved scale near ANU V3468. **D**, internal view of isolated scale (piece h3). **E**, isolated fang (piece h4). **F**, piece g5 showing weathered left vomerine fang of paratype, ANU V3468 (all bone and rock whitened with ammonium chloride, except E, unwhitened).

**Figure 21:**
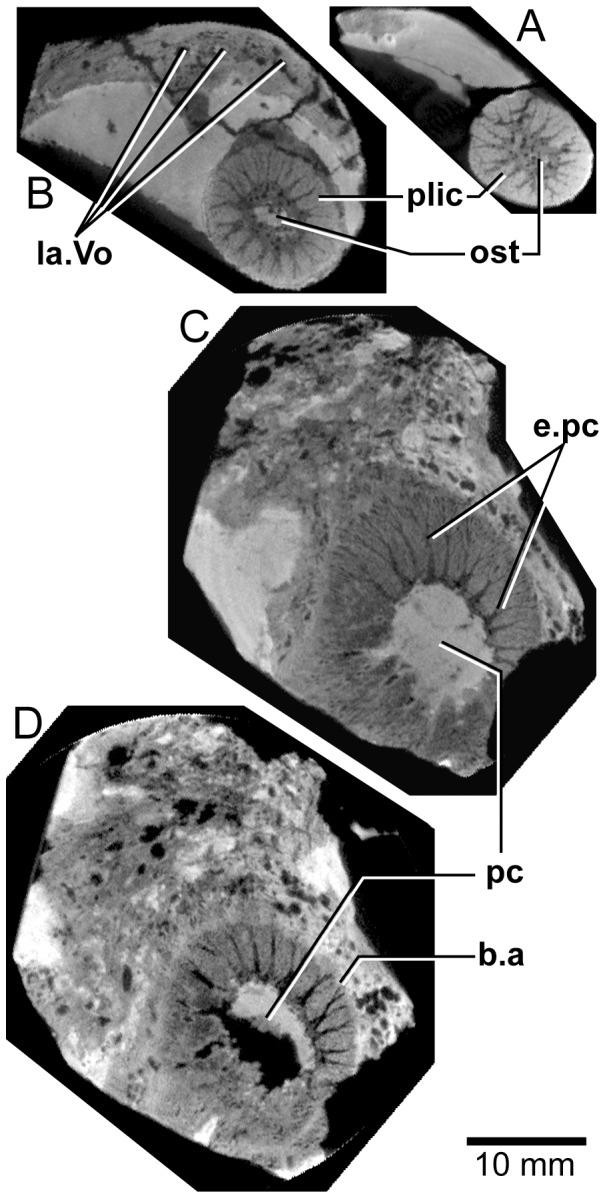
*Edenopteron keithcrooki* gen. et sp. nov. Holotype (ANU V3426). Four spaced sections through XCT-scanned portion of left vomer from near the tip (**A**) to the basal attachment of the fang (**D**).

**Figure 22:**
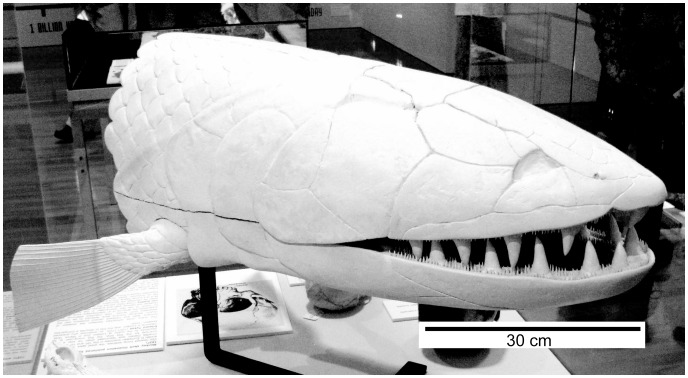
*Edenopteron keithcrooki* gen. et sp. nov. Three-dimensional model at life size (on display at Canberra Museum and Gallery, December 2011) used as a basis for the reconstructions of Figure 23A–C.

**Figure 23:**
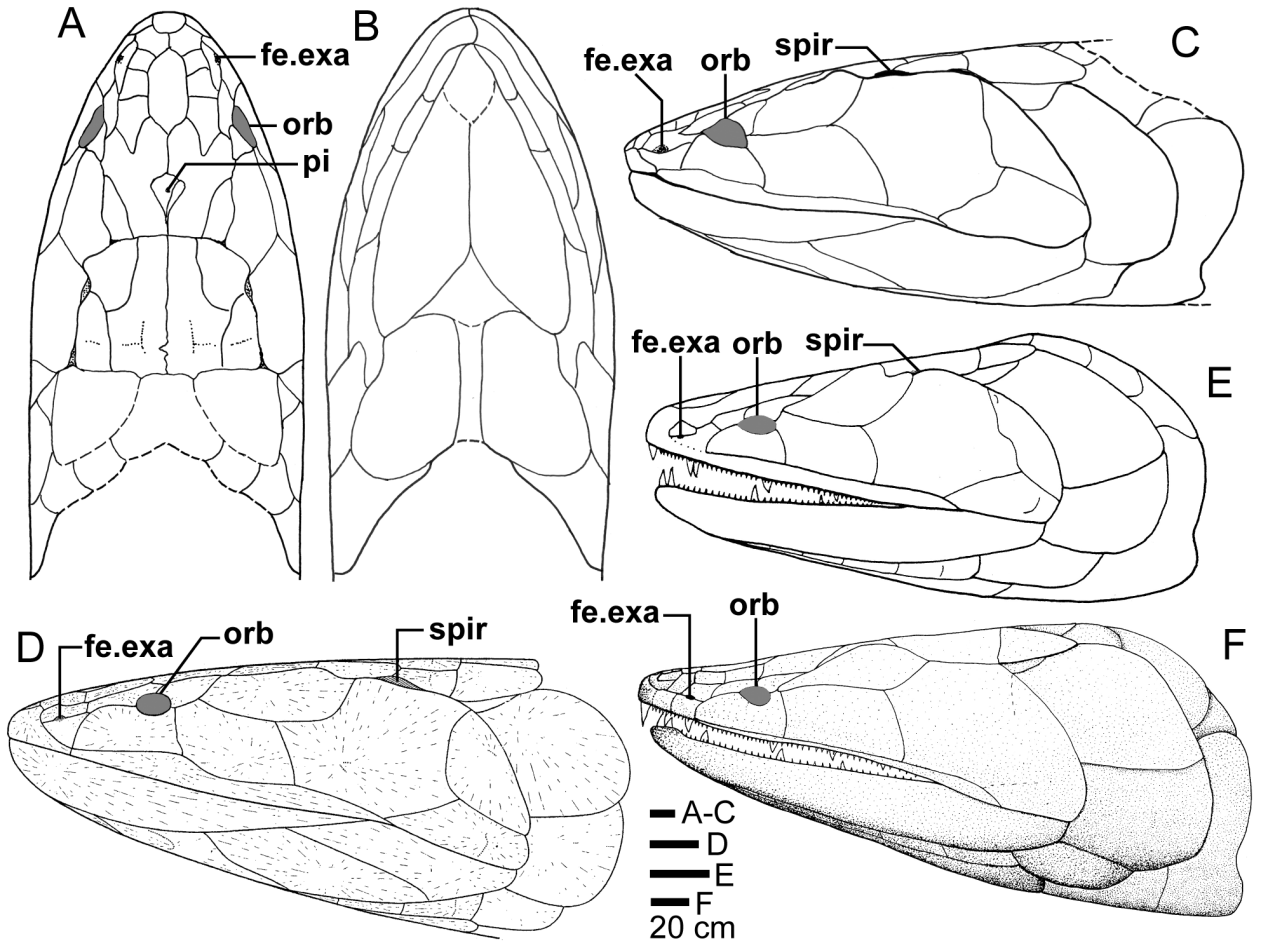
Skull and shoulder-girdle restorations. **A–C**, *Edenopteron keithcrooki* gen. et sp. nov. Restoration of head and shoulder-girdle in dorsal (**A**), ventral (**B**) and left lateral (**C**) views (based on the 3-D life-sized model shown in Fig. 22). **D**, left lateral view of *Eusthenodon waengsjoei*, shoulder-girdle omitted (after [19]: fig. 26A). **E**, left lateral view of *Cabonnichthys burnsi* (after [16]: fig. 15B). **F**, left lateral view of *Mandageria fairfaxi* (after [15]: fig. 21b).

### Name

From the nearby town of Eden, NSW, and *pteron* (Greek) wing or fin. The specific name acknowledges the contribution of geologist Dr Keith Crook (Australian National University, Canberra), who in the 1960s instigated a student mapping program on the NSW south coast that lasted over three decades, and led to the discovery of numerous fossil vertebrate sites in the Devonian rocks of the Eden–Pambula district [22], [35].

### Diagnosis

Very large tristichopterid with skull roof length (excluding extrascapulars) about 30 cm and lower jaw length about 48 cm. Endoskeleton largely or completely unossified. Parietal shield about 1.7 times length of post-parietal shield. Orbits subtriangular rather than oval; anterior supraorbital with only short slightly concave orbital margin, and pointed anterior margin; both supraorbitals slightly T-shaped. Posterior nasals with deep embayment into parietals; anterior postrostral 75% as broad as long; lateral rostral trapezoidal. Parietal in contact with postorbital bone of cheek; posterior supraorbital excludes postorbital from orbit; jugal reaches orbital margin; preorbital division of lachrymal very short; maxilla bar-like with anterolateral process. Vomer with concave anterior margin and posterior process about 54% length of parasphenoid; parasphenoid set into palate with flat to slightly convex denticulate surface; dermopalatine about 68% length of ectopterygoid; length of opercular about 66% its height; subopercular about 70% length of opercular. Posterior submandibular mainly on ventral surface, with anterior point on mesial side. Cleithrum with expanded dorsal margin, extensive postbranchial lamina, triangular ventral lamina with straight to concave posteromesial margin, and no midline contact with opposite cleithrum; supracleithrum subrectangular. Vomerine fang histology showing bifurcating pulp cavity extensions between folds of plicidentine.

### Holotype

ANU V3426, comprising an incomplete skull roof, snout, palate, both cheeks and lower jaws and associated dermal bones, left shoulder girdle and various scales. The specimen is partly compressed with some bones displaced.

### Referred Material

ANU V3468 (paratype), situated on the large block (piece h8) with *Remigolepis* (ANU V3470) and on pieces g4, g5, g9, comprising a well preserved left cheek and lower jaw in articulation, and left vomer showing large fang broken through the middle; ANU V3478, next to the previous specimen (Fig. 2A), comprising the left side of a fragmented skull roof with cheek attached (f4, h1 external impression; f2 internal impression), also associated vomerine fangs and fragmented bone and scales assumed to belong to the same individual (g2, h2, part and counterpart), and pectoral fin elements (f3, f4); ANU V3479, a left cleithrum (pieces a4, b1, part and counterpart), slightly smaller than the cleithrum of the holotype, and clavicle (pieces a1, 2), both adjacent to the parietal shield of the holotype, and associated jaw portions including a weathered fang (piece a3) and an adductor fossa steinkern (pieces a5, 6), very incomplete but presumed to belong to the same individual.

### Remarks

The large size of *Edenopteron keithcrooki* distinguishes it from other much smaller tristichopterids, the only taxa that may have reached comparable size being *Eusthenodon*, *Platycephalichthys*, *Jarvikina*, *Hyneria*, *Notorhizodon*, *Mandageria* and *Langlieria*. The dermal ornament of reticulating coarse ridges and rare tubercles is similar to that of *Eusthenodon*, but *Edenopteron* differs in numerous features including the following: proportions of skull roof (relatively shorter parietal compared to postparietal length) more elongate anterior postrostral with anterior ossification center, strong posterior nasal processes indenting the parietals, shape of posterior supraorbital, jugal reaching the orbit, very short pre-orbital part of lachrymal, quadrilateral rather than triangular lateral rostral, long low maxilla, proportions of opercular, shape and ornament of subopercular, less coarse ventral ornament (gulars, clavicle), overlap for submandibulars on the lower jaw, and scale ornament. *Platycephalichthys* also has similar dermal ornament; a single fang on the posterior coronoid, a long low maxilla with an anterior process, and possibly a dorsally expanded cleithrum may be shared with *Edenopteron* (absent or unknown in *Eusthenodon*). However *Platycephalichthys* lacks tusks on the premaxillae, had endochondral ossification, and the ornament differs in detail, as does the skull roof pattern. *Jarvikina* also differs in ornament, shape of the vomers, and endochondral ossification. *Hyneria* has similar but more reticulate ornament, probably a more elongate parietal shield, the jugal does not reach the orbit, the gulars are very elongate, and the shape of scales is characteristic of this taxon. *Notorhizodon* has similar ornament, but marginal teeth are larger, its entopterygoids had much coarser denticulation, the parasphenoid is not depressed into the palate, and the braincase was well ossified. *Mandageria* differs inter alia in its finer dermal ornament, more pointed snout, smaller orbits, shape and ventral denticulation inside the lateral rostral bone, well ossified endoskeleton and smaller scales with closer ornamental grooves. *Langlieria* also differs in its finer dermal ornament, more elongate parietal shield, more prominent premaxillary tusks, shorter posterior processes on the vomers, shape and proportions of the subopercular, and scale ornament.

## Description of *Edenopteron keithcrooki* gen. et sp. nov

The endoskeleton of this new taxon (neurocranium, jaw cartilages, gill arches etc.) was evidently mostly or completely unossified, as nothing has been preserved. The following description is based only on the dermal skeleton.

### Dermal Bones of the Skull Roof

#### Parietal shield

Latex casts of the two portions of the skull roof (parietal and postparietal shields), displaced from each other in the rock (Fig. 2C), are illustrated in approximate life position in Figure 3. The parietal shield (incomplete anteriorly) is preserved in part (piece a4) and counterpart (piece a11), the former (external impression) showing characteristic derived tristichopterid features (Fig. 3) such as the shape of the parietal and intertemporal, and the tear-shaped pineal complex close to the posterior margin. There is a small subsidiary pineal plate on the right side, presumably an individual variation as documented for *Eusthenodon* ([36]: fig. 38), although that form does not show the same pattern. Interestingly, an almost identical pineal configuration to the holotype occurs in a specimen of *Cabonnichthys* ([16]: fig. 4B). The pineal opening of the holotype was evidently lost in the saw-cut. The left intertemporal (IT) is slightly displaced, showing a mesial overlap area (od.IT), with another on the posterolateral corner (od.Po). Both sides show a clear overlap for the posterior supraorbital (od.So2), that straddles the suture between the parietal (Pa) and the anterior supraorbital (So1). A short margin in front of the pointed anterior end of the intertemporal (forming a distinct notch in the right lateral skull margin), demonstrates contact between the parietal and the postorbital of the cheek, as in both *Eusthenodon* and *Mandageria*, but unlike *Cabonnichthys*. The posterior part of a large median postrostral (Ptrp) and adjacent bones of the nasal series (Na) are preserved in position. The central part of the skull in front was lost to weathering on the surface of the outcrop, but the left anterolateral skull margin around to the midline is separately preserved in a set of small interlocking pieces, described below.

Additional information on the main shield is provided by the internal impression (a11), showing the triple junction at the posterior end of the posterior postrostral is 88 mm in front of the saw-cut, whereas it is only 48 mm in front on the external impression. This is the standard overlap relationship of these bones, but the overlap is much more extensive than in *Eusthenopteron* ([2]: fig. 14). The internal impression inside the right lateral margin is very similar to that preserved in *Notorhizodon* ([8]: fig. 22A). The point of radiation for the ossification center of the postrostral on the visceral surface is ∼12 cm from the saw-cut; in *Eusthenopteron* it is anteriorly placed at about one third the length of the bone [2], and a similar length is indicated in *Eusthenodon* ([36]: fig. 37A). Even assuming the ossification center was closer to the front of the postrostral in *Edenopteron*, it must have been more elongate than in *Eusthenodon*. This seems to be the case also in *Langlieria* Clément et al., 2009 [30] from Belgium, with a much larger median postrostral than the associated *Eusthenodon* ([37]: figs. 3B, T).

The posterior supraorbital has not been found in the holotype, even if its overlaps are clear on both sides (od.So2, Fig. 3). The bone itself is preserved in ANU V3478, which comprises a badly crushed left side of a skull with cheek attached, bisected by the main saw-cut (Figs. 11, 12). The posterior supraorbital was evidently less elongate than in *Eusthenopteron*, where it arched over the orbit. In *Edenopteron* it was positioned mainly behind the orbit, but with a somewhat different shape to the tear-shaped posterior supraorbital of *Eusthenodon*, with a slightly T-shaped form resulting from a lateral process projecting behind the orbit to contact the jugal. The anterior edge is lost in the saw-cut, but overlaps on the skull of the holotype show it must have had a triangular shape, like *Mandageria* (AMF 96855) ([15]: fig. 10a). In *Cabonnichthys* the supraorbital has a similar prominent lateral process, but at least in the holotype (AMF 96858) there was an extra mesial process giving a quadrilateral rather than triangular shape ([16]: fig. 3A). In that form it was restored enclosing most of the orbit, whereas in *Edenopteron* the orbital margin on the anterior supraorbital is clearly seen (orb.m). The lateral process of the ‘T’ on the posterior supraorbital clearly excluded the postorbital bone from the orbital margin, as in *Mandageria*, *Cabonnichthys*, and *Eusthenodon*, but not *Eusthenopteron*.

The orbital margin on both anterior supraorbitals of the holotype is only slightly concave, so the bone contributed only to the dorsal margin of the orbit. A well preserved overlap area on the right side (od.La) forms a distinct right angle, in contrast to the curved overlap area in this position for the lachrymal in *Eusthenopteron*. The lachrymal of *Edenopteron* has only a rounded anterior angle in front of the orbital notch (well preserved on the right cheek; Figs. 9, 10) so it is assumed that the anterior part of this overlap area was covered by the lateral rostral. On the counterpart the left anterior supraorbital has a well preserved margin in front of the lachrymal overlap, slightly notched where another suture seems to run anterolaterally, forming a triple junction, presumably the anteromesial corner of this bone. The right supraorbital has a pointed anterior margin, mesial to which the posterior corner of another bone is preserved, possibly the tectal (?Te), or perhaps the next element in the nasal series.

The external impression shows a clear mesial suture between the anterior supraorbital and the posterior nasal series on both sides (flattened on the left side, but retaining the lateral curvature on the right). The posterior suture of the posterior nasal is obscured by cracking, but indicates a posterior prong into the parietal on both sides, as in *Eusthenodon* but evidently more pronounced. The central skull region in front was completely weathered away on the holotype, the next preserved bone being the anterior part of the anterior postrostral (sandwiched between the premaxillae; see Figs. 4A, 5A). In the missing region, two smaller nasals and a larger pair meeting in the midline can be assumed after Jarvik’s [19] *Eusthenodon* reconstruction; there is an indication of a midline suture between nasals behind the anterior postrostral in a separate piece preserving the snout (Na, Fig. 5A).

#### Post-parietal shield

The external surface is preserved mainly on piece a11, with the right tabular adjacent to the sawn edge on piece c6, and internal surfaces preserved on pieces b5–7. This unit was ∼112 mm long. The postparietals were slightly displaced (PPa, Fig. 3), their median suture showing a slight interdigitation near the posterior margin as in some other tristichopterids (e.g. *Cabonnichthys* from Canowindra). The well preserved left supratemporal (St) shows a deep spiracular notch (spir) at its posterior suture with the tabular (Ta). In front is a distinct lateral process that indented a notch in the dorsal cheek margin, apparently more pronounced than in *Eusthenodon*, but not as marked as in some *Cabonnichthys* (e.g., AMF 96856), where it may form a distinct anterolateral projection ([16]: fig. 4B). The right tabular is relatively complete across the saw-cut but slightly flattened, with a short transverse pitline at its center (pl). Transverse and longitudinal pitlines over the postparietal ossification centers, as in other tristichopterids, are indistinct and obscured by cracks in *Edenopteron*.

The posterior margin of the post-parietal shield is incomplete and partly lost in the saw-cut. It can be interpreted after the configuration in *Eusthenopteron*, with a central part that abutted or overlapped the median extrascapular, and lateral parts with a projecting posterior lamina ([2]: fig. 14). The same arrangement is shown in *Eusthenodon* ([19]: fig. 24). For *Notorhizodon*, the median extrascapular (unknown in *Edenopteron*) suggests that posterior skull projections met in the midline ([8]: fig. 26A), perhaps approaching the condition in *Mandageria* where the median extrascapular narrows almost to a point [16]. A specimen of *Marsdenichthys* recently described (NMV 179619) seems not accurately restored ([7]: fig. 3A), as the latex cast shows a central part of the posterior skull margin which evidently overlapped the median extrascapular, with a distinct process and lateral notch where the posterior pitline, which passed across onto the lateral extrascapular, the latter bone evidently overlapping the skull. For *Edenopteron* similar processes are suggested on the holotype (Fig. 3). The postparietals are crushed and incomplete on a second specimen (Fig. 11), with the left bone displaced back against the midline suture, and the more extensive right postparietal showing a strong posterior overlap for the lateral extrascapular (od.Esc, Fig. 12).

#### Snout

Piece a8 of the holotype attaches at the front of the main palate impression (on piece b7) and preserves the internal impression of the dermal bones of the snout (Fig. 4B). Piece a8 sits ∼16 mm higher than the palate surface; both premaxillae meet in the midline (Pmx, Fig. 5), fixing the midline position of the anterior edge of the snout at about 20 cm in front of the saw-cut at the posterior edge of the parasphenoid. Noteworthy is a large tusk (t.Pmx) and circular attachment for a corresponding tusk near the midline suture between premaxillae. Premaxillary tusks are known in the Canowindra tristichopterids (*Mandageria*, *Cabonnichthys*), and various other sarcopterygians including an undescribed species of *Eusthenodon* [15], but they do not occur in *Eusthenopteron* or *Platycephalichthys*. These ‘pseudofangs’ also occur in *Langlieria* Clément et al., 2009 [30] from Belgium, and *Bruehnopteron* Schultze & Reed, 2012 [31] from Nevada. We use the terminology of ‘tusk’ rather than ‘fang’ [15], the latter reserved for large teeth in alternating replacement pairs as on the dermal palate bones and coronoids of the lower jaw. The snout of *Edenopteron* clearly had a less pointed shape than restored for *Mandageria* [23]. The premaxillary tusks sit on a thickened ridge just inside the anterior margin of the premaxilla (la.pal, Figs. 5B, 14A). Piece g2 (with preserved bone and tooth tissue on the counterpart h2; assumed to belong to ANU V3478) shows an impression of closely spaced tusks evidently folded back beneath the premaxillae (Fig. 14B). These also sit on a thickened ridge (la.pal), in front of which is a poorly preserved anterior margin of similar shape to that of the holotype. As preserved these resemble the premaxillary tusks figured for *Langlieria* ([30]: fig. 4). Behind this ridge the holotype shows a shallow v-shaped notch opening into a distinct anterior cavity (Fig. 4B), an unusual configuration because the prenasal fossa, floored by the ethmoidal ossification of the neurocranium, is usually visible in this position ([5]: fig. 5). In the *Edenopteron* holotype the floor of this cavity shows posterior radiating striations of the postrostral plate (Fig. 5B), indicating an anterior ossification center for this bone. The anterior postrostral has a central ossification center in *Eusthenopteron*, and also *Eusthenodon* ([19]: fig. 26B). The midline suture between premaxillae is clear internally between the tusks (Figs. 4B, 5B), but externally is obscured by a crack (junction of pieces a7, a14). It is assumed they met externally in front of the anterior postrostral, which shows a very clear anterior margin on the right side (Ptra, Figs. 4A, 5A). The posterior margin is less clear, but indicates the postrostral was about 75% as broad as long, whereas in *Eusthenodon* it is equilateral, and in *Eusthenopteron* it is broader than long. The triple junction between the postrostral, right nasal and premaxilla is also clearly preserved. Presumably the nasals met behind in the midline, but this region is poorly preserved (dashed line, Fig. 5A).

Piece a7 (left premaxilla) connects around the left skull margin with pieces a10 and e2 (*Remigolepis* V2378) to preserve external bone impressions for about a 20 cm distance from the midline (Fig. 7A; internal impressions preserved on a13, b6, 7). Immediately adjacent, the distinctive anterodorsal overlap of the postorbital bone (od.Pa/IT; described below) demonstrates that the left cheek was still placed against the skull, but separated and offset by a fracture. The preserved left external anterolateral margin of the skull lies ∼60 mm from the anterior preserved end of the parietal skull portion, which as noted above had been rotated anti-clockwise so that its midline is almost 90° from the palate and marginal jaw bones (Fig. 2C). In lateral view the premaxilla shows a pronounced posterior process (Pmx, Fig. 8A), slightly pulled apart from the adjacent bone, the latter with a rounded dermal process projecting anteromesially into a notch of the premaxilla (R.l, Fig. 7E). Underneath, a strong mesial lamina projecting inwards some 10 mm (pr) appears to be part of this posterior bone, although no corresponding structure is evident in restorations of the lateral rostral in *Eusthenopteron* ([38]: fig. 53). There is some uncertainty about the shape of the lateral rostral and adjacent bones in *Edenopteron*, although certain aspects are clear.

About 25 mm behind the anterior end of the lateral rostral (on piece a10) is a distinct shallow notch on the dorsal margin (nn, Figs. 7D, 8A, C), delimited behind by a smooth mesial surface projecting in at about 90° to the external surface. This notch is interpreted as lower edge of the external nasal opening. It is about 15 mm across, with the smooth mesial projection (pr.dim, Fig. 7D) about 10 mm long, and showing a patch of very fine denticulation (dent, Fig. 8C) perhaps corresponding to the special ornament inside the nasal opening of *Gogonasus* ([39]: fig. 13). Smooth bone or with very fine ornament also lines the external nasal opening and process dermintermedius of the lateral rostral bone in *Eusthenopteron* ([38]: pl. 6, fig. 2). However on the rock surface this denticulated area is separate from the lateral bone impression, so our interpretation is provisional, although the denticles are clearly seen to be separate from the adjacent impressions of scales (sc, Fig. 7A).

About 20 mm behind this nasal notch, a clear bevelled margin with a raised rim ∼15 mm long could either be the orbital margin of the lachrymal (Fig. 7A), or defining the upper (mesial) margin of an elongate lateral rostral (Figs. 7D, 8C). The latter interpretation gives a completely different shape to the lateral rostral of *Eusthenodon*, well preserved in the holotype ([19]: pl. 10), and demonstrating a morphology and triangular shape very similar to that of *Eusthenopteron*. In *Edenopteron* the lateral margin beneath the nasal notch forms a shallow embayment with fine rugose ornament extending back past the next crack (onto piece e2), and the preservation suggests that not much is missing (Figs. 7D, 8C). Alternatively, if part of the ornamented surface represents the lachrymal, bounding the orbit ventrally, there is no sign of a suture in the correct position (dashed line, Fig. 8A). A shallow sulcus of unknown function (sulc) just lateral to the nasal notch is in the wrong position to be part of the suture between lachrymal and lateral rostral. The adjacent ornamented area (∼25 mm wide), is narrower that the corresponding part on the much better preserved right cheek (Fig. 9A), so could be incomplete. The rock surface shows no clear edge, with slickensides on the surface where the bone impression grades into matrix, so depth of the lateral rostral as restored (Fig. 8C) may not be reliable.

Behind the level of the orbit (obscured by fractures) the bone surface of the left cheek is stepped down across a fracture onto piece a12, and evidently displaced forward. The distinct dorsal overlap belonging to the postorbital (od.Pa/IT, Fig. 7A) suggests that the region behind must include part of the squamosal, and below part of the jugal (Fig. 8A), but fractures are difficult to distinguish from sutures. A long opening crossing the bone surface behind the dorsal overlap area of the postorbital (Fig. 7A) suggests a suture or perhaps breakage along a sensory canal, but if natural is difficult to interpret in this position.

A small bone positioned immediately in front of the right supraorbitals of the rotated skull roof (Te, Fig. 7A) is interpreted as a displaced tectal, presumably also from the right side. Preserved length is 30 mm and maximum preserved width (behind the ventral notch) is 13 mm. A short section of the mesial margin is preserved about 7 mm above the nasal notch (nn, Figs. 7B, 8B). The latter is 15 mm long and 6 mm deep, corresponding in size to the notch of the lateral rostral, and implying a nasal opening (fe.exa, Fig. 23) considerably larger than in *Eusthenodon* (5 mm long by 3 mm high in the holotype ([19]: pl. 10). However that specimen was less than half the size of the holotype of *Edenopteron*. A ventral view (Fig. 7C) shows an expanded rounded thickening just behind the notch (th), inside of which a smooth concave surface continues upwards and anteromesially (pr.te), presumably the tectal process leading to the nasal cavity ([38]: fig. 53). Anterior and posterior margins of the tectal are missing, but it seems the nasal notch was relatively larger for the size of the bone than in *Eusthenopteron*, although positioned near the anterior end as in that form, and *Eusthenodon*. As restored it is more elongate than the rather equilateral tectal in the restorations of *Mandageria* ([15]: fig. 21).

Jarvik’s [2], [5] restorations of *Eusthenopteron* show the infraorbital sensory canal passing via the lateral rostral to the premaxilla, but in *Bruehnopteron* from Nevada [31], and re-studied specimens of *Eusthenopteron* from Miguasha, it passes directly from the lachrymal to the premaxilla, as is the case also in *Jarvikina* and *Platycephalichthys* (H.-P. Schultze, pers. comm. 17 Feb 2012). The restoration of *Mandageria* shows a lateral toothed margin on a broad lateral rostral ([15]: figs. 6c, 21b); this would require the infraorbital canal to pass through it, but according to P. Ahlberg (pers. comm., 4 July 2012), the teeth are carried on the premaxilla passing back inside the lateral rostral. The displaced maxilla in our specimen (see below) displays an ornamented surface right to the anterior tip, so the lateral rostral must have been excluded from the jaw margin, the normal arrangement in other tristichopterids apart from *Mandageria*.

As noted above, the relation between the premaxilla and the preserved palate indicates the anterior edge of the snout was about 20 cm of midline length in front of the posterior edge of the parasphenoid, or about 17.5 cm in front of the presumed position of the buccohypophyseal foramen (f.bhp, Fig. 5B). In *Eusthenopteron*, based on the restored neurocranium ([5]: fig. 11B–C), the center of the pineal cavity is 28% of total length of the anterior moiety (ethmosphenoid) anterior to the level of the buccohypophyseal foramen. In *Notorhizodon* the level of the buccohypophyseal foramen is about 47 mm in front of the posterior edge of the parietal bone, and approximately level with the anterior corner of the intertemporal, which on the skull pattern of *Edenopteron* would place it slightly in front of the pineal plate; i.e. reversing the situation of *Eusthenopteron*, which could be attributed to posterior migration of the pineal relative to the length of the snout. In the restored palate of *Notorhizodon* ([8]: fig. 37A) the buccohypophyseal foramen is well in front of the adductor fossa, whereas for *Eusthenodon* it was reconstructed only slightly in front, level with the posterior end of the ectopterygoid ([19]: fig. 29). Thus the previous restoration of *Notorhizodon* could be adjusted, but the buccohypophyseal foramen is approximately level with the anterior fang of the ectopterygoid, as seems to be the case also in the *Edenopteron* palate (Figs. 4B, 5B). The buccohypophyseal foramen of *Edenopteron* lies close to the level of the anterior end of the ectopterygoid (but there was perhaps some displacement), and about 9–10 cm in front of the adductor fossa (as preserved on the right side).

### Dermal Bones of the Cheek and Palate

#### Cheek unit

The right cheek of the holotype (Fig. 9) is preserved in part and counterpart on numerous pieces (internal surface on c1, c2, d4 behind saw-cut, b4, b5, b8 in front; external surface on a11, b1, c3, some bone of the squamosal embedded in resin on c10). As noted above, the left cheek (Figs. 7A, 8A) is less informative due to crushing; possibly part of its inner surface is preserved on b6, with numerous fragmented impressions showing external ornament on b8 and c12. The overall configuration of the cheek unit is best indicated by the right inner surface. The cheek is also preserved on the paratype (ANU V3468) and incompletely on ANU V3478 (Figs. 11, 12, 13).

The inner surface of the holotype right cheek (Fig. 9B) shows the lachrymal ossification center placed close to a gentle embayment in the ventral margin (La, Fig. 10B), inside which is an internal thickened ridge (ri), presumably underlying the infraorbital sensory canal, which thus ran just above the suture with the maxilla, its normal position. The ventral ridge continues across the slightly displaced suture with the jugal (Ju). The posterior suture of the jugal is obscured by a large crack ventrally, but is clearly inferred higher up from the radiating striations of the squamosal; its dorsal suture with the postorbital (Po) is cracked, and best located on the external surface. The smooth internal surface of the jugal shows radiating striations only on the ventral ridge; these indicate an ossification center very close to the ventral margin. The jugal clearly reached the margin of the orbit. The large postorbital (Po) has much of its outer margins obscured by fractures, but a prominent dorsal process is well preserved (pr.psp, Figs. 9, 10). In life this overlapped the posterior corner of the parietal shield (od.Po, Fig. 3).

The squamosal (Sq) has a rounded dorsal margin, showing striations radiating from the ventral ossification center, placed just above the ventral ridge which is thickest at this point. The ossification centers for the lachrymal, jugal, and squamosal all seem more ventral in position than restored for *Eusthenodon* ([19]: fig. 27). A triangle of bone still attached to the rock shows the external bone surface on the cast (Fig. 9B), the break representing the suture between the squamosal and preopercular, behind which the striations have a completely different orientation (Pop, Figs. 9B, 10B). There is an internal thickening inside the posterodorsal margin of the preoperculum (th), and the broken edge shows the squamosal overlapping the preoperculum in the dorsal part of their common suture, as in *Eusthenopteron* ([2]: fig. 9). The external surface of the squamosal (on piece c3) is retained as bone, ∼3 mm thick ventrally, and nearly 8 mm thick on the ventral ridge at the squamosal ossification center. The junction with the quadratojugal (Qj) is unclear, but like *Eusthenopteron* it suggests an extensive overlap area on its dorsal margin for both the squamosal and preoperculum. Radiating striations from the posteroventral preserved corner (Figs. 9B, 10B) represent the quadratojugal ossification center. The ventral edge of the quadratojugal is preserved adjacent to the maxilla on piece d1. Its posterodorsal margin runs down to the edge of the cast, inside which is a prominent internal ridge (qj.ri).

The external surface of the cheek unit is completely prepared out in front of the saw-cut (Fig. 9A), but behind much of the bone remains, although radiating striations and some bone sutures are clear. The well preserved ventral edge across the lachrymal and jugal shows a smooth zone 12–20 mm wide right along the margin (sm). The posterior edge of the lachrymal (La, Fig. 10A) is obscured by the fractured lower part of the jugal (Ju), which is displaced forward above it. The orbital margin of the lachrymal is completely preserved as an embayed and thickened edge between anterior and posterior angles (orb.m). The anterior margin of the lachrymal is missing its middle part, but clearly was much steeper than in either *Eusthenopteron* or *Eusthenodon*, in both of which the lachrymal had a different shape, with about 50% of the length of the bone in front of the orbital margin. The fractured jugal is a little displaced forward over the lachrymal, displaying its anterior margin as a rounded edge with a slight ventral notch and process, like *Eusthenodon* and *Eusthenopteron* (the process conveying the infraorbital sensory canal). In *Eusthenodon* the jugal reached the orbit internally, but the narrow orbital margin had an external overlap for the posterior supraorbital ([19]: fig. 27A); this is absent in *Edenopteron*. The upper suture with the postorbital (Po) is very clear, running back across the saw-cut (Figs. 9A, 10A), where radiating eroded bone shows a clear triple junction with the anterior edge of the squamosal (Sq). A distinctive broad dorsal overlap (od.Pa/IT) slid under the edge of the parietal shield when the cheek was in position, extending anteriorly into the orbit where it was overlapped by the posterior supraorbital (od.So2). This relationship is demonstrated on ANU V3478, a badly fractured specimen showing the left cheek slightly displaced from under the lateral edge of the skull (Fig. 11). The dorsal overlap of the postorbital (od.Pa/IT, Fig. 12) is slightly pulled apart from under the edge of the incomplete parietal in front (Pa), and intertemporal (IT) behind. The postorbital (Po) and jugal (Ju) both show clear patches of pores indicating the passage of the infraorbital sensory canal through these bones (p.ioc). The lachrymal (La) is crushed and incomplete in front of the jugal, which shows a ventral process on its anterior edge as in the holotype. The dorsal overlap of the postorbital in the holotype terminates anteriorly at the level of the jugal-postorbital suture, with a narrow selvage (od.Ju, Figs. 9A, 10A) where the jugal projected into the orbital margin, as is clearly demonstrated in the second specimen with the jugal still in position (Ju, Figs. 11, 12). The posterior extremity of the dorsal overlap is clearly seen across the saw-cut in the holotype. *Eusthenodon* had a similar overlap ([19]: fig. 27A), but somewhat different in shape (less embayed posteriorly, reducing to a point anteriorly) compared to *Edenopteron*.

Behind the postorbital the squamosal is defined mainly by radiating striations in the preserved bone; the preopercular and quadratojugal are very poorly preserved on this specimen, but more clearly seen on the paratype (Fig. 13A). This is a crushed associated left lower jaw and cheek unit (external surface on piece h8; internal cheek g5; lower jaw internal g4 and steinkern of adductor fossa g9). It is very close in size to a right *Eusthenodon* cheek (specimen P1480) figured by Jarvik ([19]: pl. 20). Unlike that specimen the maxilla has not remained with the cheek, being displaced inwards (see below). The lachrymal and jugal as far as preserved in ANU V3468 compare closely with the holotype. The postorbital is badly crushed dorsally, but the squamosal and quadratojugal clearly show their common suture, the latter reducing to a point anteriorly as in *Eusthenodon*, rather than forming a truncated edge contacting the maxilla as in *Eusthenopteron*. The preopercular is slightly displaced to reveal the posterodorsal overlap of the quadratojugal (od.Pop), again as in *Eusthenopteron* ([2]: fig. 9). Like the sqamosal in front, its dorsal margin is unclear due to fracturing.

In each of the cheek units just described the maxilla is displaced or missing, perhaps due to less complex overlap relationship compared to other tristichopterids, for example the unique overlap area for the squamosal in *Eusthenodon* ([36]: fig. 37C). However the almost complete right maxilla of the holotype lies adjacent to the lower jaw (Mx, Figs. 15, 16). Its anterior end is well preserved on piece b2 (internal surface; Fig. 4C), the external surface running back across the larger piece carrying the jaw symphysis (b8), then across b9 and the saw-cut onto d1 (external impression) and c1 (internal impression). Including 2–3 mm at both ends, and the saw-cut thickness (∼8 mm), the complete maxilla was at least 195 mm long (Fig. 14C). Its anterior end shows a clear dorsal process (dp.Mx, Fig. 4C), a structure well documented in *Eusthenopteron*, but said to be absent in *Eusthenodon* [36]. However, two isolated maxillae from the Famennian of Grenfell, NSW, which show this process, have been assigned to ‘*Eusthenodon* cf. *wangsioi*’, the Greenland species [18]. Posteriorly, the maxilla of *Edenopteron* does not expand like in *Eusthenopteron*, nor does it have the more complex dorsal margin of *Eusthenodon* (Fig. 14C). Most of the ventral margin shows fine pointed teeth, about 2 mm of length visible externally, but longer internally (5–6 mm; Fig. 14D), with a base ∼2 mm wide. By comparison the marginal teeth of *Notorhizodon* are more robust and widely spaced ([8]: fig. 23A–B). The dorsal surface of the *Edenopteron* maxilla curves gently upward before a notch for the overlap for the jugal (od.Ju, Fig. 14C), set in about 7mm from the ornamented surface (dorsal edge not complete). The maximum depth (∼18 mm) is ∼70 mm from the front, and the bone decreases in depth posteriorly (∼12 mm deep 40 mm from the posterior end). It tapers posteriorly as in *Eusthenodon* [36], and also anteriorly, where it curves slightly downwards apparently to a point (extremity missing; Fig. 14C), rather than curving upward or with an anterior truncation (as in *Eusthenopteron* and the Grenfell examples). One specimen of the Greenland *Eusthenodon* indicates from overlaps for the maxilla that it reached back to just project beneath the anterior edge of the quadratojugal ([19]: fig. 28). There is a more extensive contact between these bones in *Eusthenopteron* [2]. About 25 mm outside the posterior preserved tip of the maxilla in the *Edenopteron* holotype is the ventral edge of the right quadratojugal, with an embayed thin margin and clear suture just anterior to the end of the maxilla. This suggests the same arrangement as in *Eusthenodon*. We did not locate the left maxilla (possibly crushed or obscured beneath the preserved palate).

The paratype (ANU V3468, Fig. 13) also preserves the left maxilla, showing the same bar-like shape. Both premaxilla and maxilla are dislodged down inside the dentary, the former preserved in two portions displaced across a joint in the rock. The maxilla is preserved in three sections, the anterior with a broken anterior edge, and the posterior showing a dorsal overlap (od.Ju, Fig 13B), and again reducing in height to a posterior point at about the same level as the posterior end of the dentary on the lower jaw, which would thus have excluded the squamosal from the jaw margin.

A ‘bar-like maxilla’ was stated as a unique distinguishing feature of *Marsdenichthys* [7], but the above description and comparisons indicate that this is not a reliable character.

#### Palate

Piece b7 (Fig. 4B), the largest preserved portion, displays the entire denticulate part of the parasphenoid, part of both vomers, dermopalatines, entopterygoids, and the left ectopterygoid. The almost complete right vomer, including the fang, has its anterior edge preserved on a8, and lateral part on b3. The left vomer on a9 includes a complete fang (at least 41 mm long measured from its root), its posterior process extending onto b7. The left ectopterygoid, entopterygoid and dermopalatine are most complete. The right side continues across the saw-cut onto c1, where ‘steinkerns’ of both adductor fossae are preserved in articulation (Fig. 17A).

The posterolateral edge of the left vomer is very clear (Fig. 4B). It is more convex than in *Eusthenopteron* or *Eusthenodon*, but similar to this margin in *Platycephalichthys* ([40]: fig. 23). Its overlaps with the anterior edge of the dermopalatine and entopterygoid are pulled apart (od.Vo, Figs. 4B, 5B). The denticulate part of the entopterygoid (Ent.tp) stands up with a laterally directed ridge, its surface covered with scattered fine dentition (best seen where bone adheres to the impression on the rock surface, showing that ornament was finer than in *Notorhizodon*). *Notorhizodon* also differs in its stronger ‘labial ridge’, which projects prominently towards the labial margin (r.lab, [8]: fig. 30), rather than out (downwards) from the denticulate surface as shown by *Edenopteron*.

The vomer of *Hyneria* was said to lack posterior processes [33], but more recently it has been stated that they are ‘at least 45%’ the length of the parasphenoid [41]. In *Edenopteron* the posterior processes are ∼54% parasphenoid length (ppr, Figs. 4B. 5B).

The parasphenoid (Psp, Figs. 4B, 5B) has a sharp anterior point, is widest (∼30 mm) just behind the level of the posterior processes of the vomers, and narrows posteriorly, being slightly waisted about 25 mm in front of the posterior margin, at the assumed level of the buccohypophysial foramen (f.bhp), with posterior bone radiations behind this level. In *Notorhizodon* this part of the parasphenoid is expanded posteriorly. The ventral denticulate surface in *Edenopteron* (not well preserved) is flat to slightly convex (with a depression at the ossification center); clearly it was different to the concave and broad shape of *Notorhizodon*. The left side at the back end indicates an upward projection (asc.pr, Fig. 5B). The left entopterygoid is pushed down beneath the edge of the parasphenoid, from which it is separated by matrix. On the right side a faint lineation represents the margin of a separate element corresponding to the ‘accessory vomer’ in *Mandageria* and *Cabonnichthys* (ac.Vo), which is more clearly seen on the counterpart (see below).

Both dermopalatines carry remains of one large fang (f.Dpl), the right with a base ∼20 mm in diameter (and the tip of a second fang preserved behind it). The left dermopalatine shows clearly the mesial margin pulled away from the entopterygoid, which according to Jarvik ([2]: 37) fitted into a groove. A slight but distinct mesial angle opposite the main fang notches the left entopterygoid (n, Fig. 5B). The remnant of the second dermopalatine fang was probably just erupting when the animal died. The left dermopalatine shows the contact face where the vomer has pulled away slightly at the front, a clear posterior margin where it pulled away from the entopterygoid, and the fang positioned more to the posterior, without any sign of a second fang or pit. The overall shape of the dermopalatine is similar to *Eusthenopteron*, and it measures about 70 mm long. The posterior margin shows no evidence of the complex interfingering on this suture seen in *Notorhizodon*. Although no tooth row is evident on the cast, the specimen reveals teeth within the non-removed bone, and on the sawn edge through the right dermopalatine a vertical bony lamina projects ∼12 mm downwards; i.e. as illustrated by Jarvik ([38]: fig. 55). The opposite side of the saw-cut shows a tooth projecting from the lamina (total depth ∼ 20 mm). A thickened anterolateral ridge preserved on the left dermopalatine (ri, Figs. 4B, 5B) must represent the inner edge of the choana, as in *Eusthenopteron*, but shows no indication of a tooth row on this part of the ridge.

Sutures between the entopterygoid, ectopterygoid, and dermopalatine are clear on the left side. The left ectopterygoid fang is near its anterior margin, with a second smaller fang at the preserved edge of b7 (f.Ect, Figs. 4B, 5B). The right ectopterygoid is behind the saw-cut on piece d7 (dermopalatine suture lost in the saw-cut), and continues back as steinkerns of the adductor fossa on piece c1 (Fig. 17A). This shows the root of a large fang on the saw-cut, and a posterior fang with a strong vertical lamina partly exposed through the rock matrix about 55 mm behind the first (the same distance between fangs on the left ectopterygoid). The upper and lower adductor fossae come together (anterior edge) about 48 mm behind this, suggesting an ectopterygoid about 116 mm long, if it reached the anterior edge of the adductor fossa as it does in *Eusthenopteron*. In *Notorhizodon* a smooth lamina (lv.Ent, 8: fig. 30] separated the adductor fossa from the denticulate part of the ectopterygoid, but this detail is not shown in *Edenopteron*. *Mandageria* is reconstructed with the entopterygoid excluding the ectopterygoid from the adductor fossa [23], and the dermopalatine is only slightly shorter (98%) than the length of the ectopterygoid, whereas in *Eusthenodon* it is about 83% the length, and in *Eusthenopteron* it is considerably shorter (64%). In *Edenopteron* this proportion was about 68%.

A composite latex of the counterpart (pieces b5, b6, a11) shows the upper surface of the dermal bones of the palate (Fig. 6). The parasphenoid anterior tip (visible in Fig. 3) is more oblique, the whole element having a different shape to its ventral outline. It is more elongate (preserved length 157 mm), and gently concave over most of its surface, deepening to a spatulate shape at the posterior end where a central depression evidently housed the hypophyseal fossa (fo.hyp, Fig. 6B). Parts of both vomers are in position, the more complete right vomer extending from the incomplete anterior margin (deeply embayed with a marginal ridge), back to the posterior process (ppr) some 82 mm behind. A roughened surface marks the fang position and ossification center, with clear radiating striations to the preserved extremities of the bone. Both vomerine fangs are in position on pieces b7 and a9, the latter XCT-scanned to show the presence of a tooth-bearing lamina (see Fig. 21). In anterior view the fangs (∼45 mm long) curve inwards, their tips ∼33 mm apart, with the center of bases ∼40 mm apart. The 2 mm saw-cut (Fig. 2A) exposes 30–40 mm thick bone, and a toothed lamina of the left vomer. The vomer anterior margin is not completely exposed, but rather than the transverse edge of *Eusthenopteron* or *Jarvikina*, it was evidently more deeply embayed (riVo, Fig. 6B), as in *Eusthenodon* ([19]: pl. 16).

Both entopterygoids were displaced upwards by compaction, such that their mesial edges (Ent, Fig. 6A) curve up above the level of the parasphenoid. On the right side of the parasphenoid, mesial to the entopterygoid, a separate bone is preserved which must be the accessory vomer (ac.Vo, Fig. 6B). It has a clear lateral margin as a rounded edge, and a sinuous central ridge posteriorly, which curves over to the mesial edge about midway along the bone (ri). The anterior end is pointed, with radiating striations, reaching just past the posterior process of the vomer (as in *Cabonnichthys*; in *Mandageria* the accessory vomer just reaches the posterior process). A groove inside the anterior extremity suggests a blood vessel (gr). An oblique edge with a roughened surface towards the posterior of the bone may have abutted against the ascending process of the parasphenoid. The posterior extremity of the bone is obscured by a crack. The inner surface of this bone has not previously been described. As noted above, an analogous or homologous bone is widespread in palaeoniscoids ([42]: 273).

### Dermal Bones of the Lower Jaw and Operculo-gular Series

#### Lower Jaw

In the holotype the external surface of both lower jaws are slightly splayed out, with anterior ends still together in the jaw symphysis (Figs. 15, 16). The right jaw is rolled over with the maxilla sitting on its lateral side, and its posterior end obscured by the gular. The left jaw is displayed almost to the posterior margin, where the smooth external overlap for the quadratojugal (od.Qj, Fig. 16) is just visible in front of the overlying subopercular (Sop). This represents the fourth infradentary (Id_4_, Fig. 16), with its ventral and anterior margins clearly seen. One apparently natural lineation amongst surface fractures may be an infradentary pitline (pl.Id_4_). The dorsal margin in contact with the dentary is broken. The third infradentary (Id_3_) seems of more oblong shape than restored for *Eusthenodon*, with its anteroventral suture showing a distinct inflection ventrally. An elongate overlap area along the ventral margin broadens anteriorly (odSbm), showing a slight notch (n) on the ventral margin of the second infradentary (Id_2_). Its dorsal margin crosses the saw-cut to a slight angle in two other sutures, assumed to be the ventral edge of the dentary (De). Just beneath is another possible pitline (pl.Id_2_), but the area is somewhat fractured. The ventral suture fades out to the anterior, and margins are unclear, so a suture between first and second infradentaries is not shown (Fig. 16). The right jaw being more rotated exposes its ventral edge, with a flat smooth margin broadening slightly anteriorly and posteriorly; a narrower middle part might represent the junction between the first two infradentaries (Id_1,_ Id_2_), but again no suture is evident. A longitudinal lineation may be the ventral edge of the dentary, or possibly another pitline (?pl). Radiating striations in the abraded bone suggest a similar anterior position for the dentary ossification center as in *Eusthenodon* ([19]: fig. 26A).

The inner lower jaw surface is poorly known, the coronoids and prearticular being largely enclosed by rock matrix in the holotype. There is no evidence on whether a parasymphysial plate was present, this previously suggested to be a tristichopterid characteristic [16]. The lower surface of piece b7 has the jaw symphysis and anterior 22 cm of the left lower jaw preserved as bone (Fig. 17B). Rows of small teeth along the margin (?m.dent) could be tooth rows of the coronoids. They seem too deep to be on the dentary, but preservation is insufficient to decide this. The counterpart (external impression; piece b8) shows no sign of a marginal tooth row (Fig. 15), so either they were lost in preparation, or these small teeth do represent coronoid tooth rows. Coronoid tooth rows were interpreted as primitive ([16]: 668), as they occur in *Eusthenopteron*, *Jarvikina* and *Notorhizodon*, whereas marginal coronoid teeth are absent from the anterior coronoids in *Eusthenodon*, *Mandageria* and *Cabonnichthys*, presumably representing the more derived state.

The base of the dentary fang is visible through broken bone of the left lower jaw close to the jaw symphysis on piece b7 (f.De, Fig. 17B, C). Again there is no sign of marginal teeth between the dentary fang and the jaw symphysis, this being one of two characters by which *Langlieria* from Belgium was distinguished from *Mandageria* [30]. This is a character conflict for the sister group relationship of *Mandageria* and *Eusthenodon* to the exclusion of *Cabonnichthys* [18], the marginal dentition going past the dentary fang in *Mandageria* (and also *Notorhizodon*), but not in *Eusthenodon* or *Cabonnichthys*.

About 65 mm behind the dentary fang, the base of a very large first coronoid fang (diameter ∼25 mm) is exposed in a drill-hole through the bone (f.Co_1_, Fig. 17B, C). Most of this fang has been prepared out from the rock matrix (length ∼42 mm), and shows a flattened cusp with cutting edges, in contrast to the rounded vomerine fangs of *Edenopteron*. The prearticular of the inner side of the jaw is visible only in section on the main saw-cut. Across the saw-cut both adductor fossae of the mandibular joint are preserved as bone-covered steinkerns in part and counterpart (pieces c4, d8), but these also show no sign of fangs (Fig. 17A). Noteworthy is the absence of any internal bone at the mandibular joint, the rock matrix filling the gap between thin lateral and mesial bone layers. This suggests that, as with the neurocranium, the jaw endoskeleton (quadrate-articular) was poorly ossified.

ANU V3468 also has the lower jaw preserved in articulation against the left cheek. The dentary fang impression is preserved on the main block (h8), where it is displaced ventrally across a joint (f.De, Fig. 13B). Behind this the visible fangs are as follows (measurements center to center): 60 mm behind the dentary fang is the first weathered coronoid fang, 55 mm behind this is a second fang, and 60 mm farther back is a third fang, the last being 40 mm in front of the anterior edge of the adductor fossa (all preserved on piece g4). The thickened eroded bone of the coronoids is too badly preserved to show intervening sutures. Another fang could be obscured immediately in front of the adductor fossa, but the external impression (Fig. 13) suggests that only enlarged teeth occur in this region. Thus a second fang of the posterior coronoid may not be developed in *Edenopteron*, which would be a shared resemblance with *Notorhizodon*, and some figured lower jaws of *Platycephalichthys* ([40]: pl. 17), although, as previously discussed ([16]: 667), this condition is variable within *Platycephalichthys*. The advanced condition of two fangs on the posterior coronoid occurs in *Eusthenopteron*, *Eusthenodon*, *Cabonnichthys*, and presumably *Mandageria*.

#### Operculum

The left opercular and subopercular are preserved behind the lower jaw in the holotype (Op, Sop, Fig. 15). The opercular is somewhat crushed, but partly retains its dorsoventral curvature (∼80° as preserved). Its ventral edge does not correspond to the shape of the dorsal overlap area on the subopercular, so is incomplete. However it is clear that the opercular was longer than the subopercular (Fig. 18B). The dorsal overlap is completely preserved as is the sloping posterior margin. The anterior margin (Fig. 18B) is thick and slightly convex (but less so than in *Eusthenopteron*). In overall shape the bone seems more narrow dorsally and expanded ventrally, with the posterodorsal margin as far as preserved being clearly less convex than in *Eusthenodon* [19]. This is confirmed by the more complete and uncrushed right opercular (Fig. 18A), preserved as an internal impression just above the clavicle (pieces d2, d5, d9), its external impression preserved on c6, c8, c9. With compaction of the holotype this bone was evidently compressed down onto the inside of the (displaced) left clavicle, but otherwise was not much displaced. The anterodorsal corner is missing, its preserved edge showing a depressed area presumably representing the overlap area for the postspiracular, as in *Eusthenopteron* ([2]: fig. 9B). No other evidence of the postspiracular has been found so far in *Edenopteron*. The external surface of the opercular is gently concave ventrally and gently convex dorsally, and its anterior margin changes from gently concave dorsally to gently convex ventrally. The dorsal half of the anterior margin has a smooth bevelled edge ∼7 mm wide, similar to that of the subopercular. The ventral half is also bevelled, but is thinner with ornament right to the edge except for the rounded anteroventral corner, which is smooth. The adjacent part of the external surface of the subopercular is also smooth. Measured dimensions for the right opercular are ∼150 mm dorso-ventrally and 90–95 mm antero-posteriorly. Thus it was clearly higher in proportion to length than the opercular of *Eusthenodon* ([19]: pl. 12, fig. 2) which has length about 75% its height (∼66% in *Edenopteron*). The opercular is longer than the subopercular in *Eusthenodon*, as in *Edenopteron*, but in *Eusthenopteron* it is the opposite, with the subopercular longer than the ventral edge of the opercular.

The left subopercular of the holotype is excellently preserved (Sop, Figs. 15, 16, 18B), uncrushed and little displaced above the posterior end of the lower jaw. Its dorsal edge is obscured under a displaced scale (sc) and the overlying opercular, but the shape of the overlap area is clear (Fig. 15). The external surface is slightly convex, and ornamented except for a smooth zone extending up the anterior edge to the dorsal overlap. The concave anterior margin forms a thick (13 mm) bevelled edge that abutted against the lower jaw (a.LJ, Fig. 18B). The posterodorsal extremity is missing, but maximum preserved length (∼65 mm) indicates that the subopercular was only ∼70% the length of the opercular (it is restored as slightly shorter in *Eusthenodon* [19]). In contrast, *Eusthenopteron* has the subopercular longer than the opercular ([2]: fig. 9B). The subopercular of *Edenopteron* is folded around an angle of ∼110° at its anterior edge, suggesting a relatively low position in the fish, straddling the lateral and ventral laminae of the cleithrum. The adjacent posterior submandibular (Sbm_1_) seems rather flat, and would have been mainly on the ventral surface and hence not visible in lateral view, this being a difference from the restoration of *Eusthenodon* [19]. Jarvik stated ([19]: 65) that the subopercular was ‘fairly high’ based on specimen P1473, which suggests that the subopercular was entirely on the lateral side, with the posterior submandibular inflected around the ventrolateral angle of the fish ([19]: pl. 16). A similar configuration occurs in *Eusthenopteron* [5], is demonstrated in a 3D prototype of the ANU *Gogonasus* specimen (ANU 49259), and also occurs in *Mandageria* (Fig. 19C). Thus *Edenopteron* apparently differs in this respect from all these taxa.

The subopercular is a similar size to one from Belgium that has been referred to the type species *Eusthenodon waengsjoei* ([37]: fig. 2B). That specimen differs from ours in having coarser ornament, and a distinct anterodorsal notch where the opercular overlapped, plus a ventrally thickened area abutting the lower jaw (dorsal in *Edenopteron*; Fig. 18B). The subopercular of *Langlieria* differs even more, being longer and lower, with the same anterodorsal notch, but with a prominent anteriorly directed process ([30]: figs. 2, 6).

#### Gulars, Submandibulars

The two gulars of the holotype (Gu, Figs. 15, 16) have a maximum preserved length of 170 mm (right side) and 195 mm (left side). The shape is similar to other tristichopterids (*Eusthenopteron*, *Eusthenodon*), with a curved posteromesial margin that met the clavicles, and a straight midline margin, but the latter is proportionately much shorter in *Edenopteron* (80–90 mm for left bone; cf. 120 mm for clavicular margin). Both gulars are similarly preserved, neither showing the anterior end. Their lateral margins are flat and straight to convex with a smooth edge posteriorly, and slightly concave anteriorly with a more distinct overlap area. In front of the gulars as preserved is a triangle between the jaws devoid of bone, perhaps for a median gular as restored for *Eusthenodon* [19]. The gulars are splayed apart, and could have been displaced backward. However the left gular at its posterior end is only slightly displaced from the posterior submandibular (Sbm_1_, Fig. 16), which in turn aligns closely with the subopercular (Sop), indicating little displacement from life position. By comparison with Jarvik’s *Eusthenodon* reconstruction, we could only fill the anterior triangle in the *Edenopteron* restoration by assuming the anterior ends of both gulars are incomplete.

Submandibular elements are associated with both lower jaws of the holotype (Figs. 15, 16). Some were displaced to reveal a broad overlap running along the ventral edge of each jaw (od.Sbm). Two displaced and incomplete elongate elements are adjacent to the right jaw (Sbm_2,_ Sbm_3_), the posterior element sitting over the ventral edge of the lower jaw, with the adjacent gular (Gu) slightly displaced mesially to show its smooth lateral edge. The posterior submandibular is close to its original position on the left side (Sbm_1_). A similar preservation is seen on the impression of both gulars in specimen P1473 of *Eusthenodon* ([19]: pl. 16, fig. 1). The left posterior submandibular extends back under the edge of the subopercular (Sop). Its anterior end projects to a point on the mesial side, not the lateral side as in *Eusthenopteron* [2], thus being like *Eusthenodon* [19], but more elongate and pointed. There are eight ‘branchiostegals’ in *Eusthenopteron*, but seven restored for *Eusthenodon*, based presumably on the holotype and P1483, where the left row is preserved, evidently with clearer sutures than preserved in *Edenopteron* ([19]: pl. 17, fig. 3). In *Edenopteron* these elements were more elongate and possibly less subdivided, as appears also to be the case in *Mandageria* ([16]: fig. 17). Apart from the larger posterior element (‘submandibulo-branchiostegal’ of Jarvik) there is clear evidence of only two additional elongate submandibulars (Sbm_2_, Sbm_3_), the posterior element missing on the left side to reveal the underlying overlap area (odSbm, Fig. 16).

The overlap of submandibulars in *Eusthenopteron* was fully illustrated and described [2]; they overlap the gular mesially and are overlapped by the infradentaries laterally. Thus each gular shows externally an overlap area along the lateral margin, each branchiostegal has a narrow lateral overlap, and the lower jaw in external aspect is ornamented to the ventral margin of the infradentaries. The same arrangement is seen in *Osteolepis* ([43]: fig. 24) suggesting it is primitive. In *Eusthenodon* ([19]: pls. 10, 17, fig. 3) the branchiostegals are well displayed on the holotype and a second specimen (P1483), the latter giving the best example of the gular plate. Overlap areas are not exposed, but a third specimen (P1473) shows internal impressions of the thickening at the lateral margin of the gular underlying the overlap area, with the most posterior submandibular still in position ([19]: pl. 16, fig. 1). The ventral margin of the lower jaw is not clearly illustrated in an isolated example, but Jarvik’s ([19]: fig. 27B) reconstruction reasonably assumes the same condition as previously established for *Eusthenopteron* [2].

The holotype of *Edenopteron* shows the reverse of this condition, with a clear external overlap area reaching 15+ mm wide along the ventral margin of the left lower jaw (odSbm). The same overlap relationship is also seen in ANU V3468 (Fig. 13), and is evident in some *Mandageria* specimens [15], where the submandibulars are displaced away to reveal the overlap along the ventral jaw margin (odSbm, Fig. 19C). Another example showing a broad overlap on the ventral jaw margin (“groove for submandibular bones”) is a specimen from Belgium referred to *Eusthenodon waengsjoei* [37].

### Dermal Bones of the Shoulder Girdle, Extrascapular Series

#### Cleithrum

The left shoulder girdle of the holotype (cleithrum, clavicle) is exposed in external view (pieces d2, d9) between the splayed-out jaws (Figs. 15, 16). The cleithrum is fractured but still preserves the inflection around a slightly obtuse angle onto its ventral lamina (Fig. 19B). About 190 mm of the lateral lamina is exposed externally. Its dorsal edge is partly obscured by the adjacent submandibular and gular, but is at least 70 mm wide at its dorsal preserved margin. The anterior preserved edge (just behind the posterior corner of the gular plate; Figs. 15, 16) clearly shows that breadth was expanding dorsally, in contrast to the subparallel shape of the lateral lamina of the cleithrum in *Eusthenopteron* [3], [44]. The internal impression (on piece c6) continues across a crack to piece c7, with the dorsal margin indistinct, but indicating lateral lamina length of at least 210 mm, and width of about 50 mm halfway up the lateral lamina. An incomplete cleithrum of *Platycephalichthys bischoffi* also suggests an increase in width dorsally, but the edges of that specimen are broken, and as illustrated the ornament is less linear than in our material ([40]: fig. 17). On the second cleithrum of *Edenopteron* (ANU V3479) the dorsal margin is also missing, but the ridged ornament diverges dorsally, suggesting a similar expanded shape. It is noted that a much smaller tristichopterid cleithrum from a lower level in the NSW South Coast Devonian sequence also shows a distinctly broader dorsal margin on its lateral lamina ([22]: fig. 3a).

External ornament on both cleithra of *Edenopteron* is distinctive, comprising elongate ridges and grooves generally aligned along the cleithrum, becoming finer and more meandering towards the anterior representing the postbranchial lamina. This is much better developed than in other tristichopterids (pbl, Fig. 19B). The ventral lamina is more elongate (only 40 mm wide to the anterior end of the ventrolateral angle), with a different shape in ventral view to that of *Eusthenopteron* ([5]: fig. 6C). The well-preserved posteromesial margin is straight to slightly concave, lacking the posteromesial corner of *Eusthenopteron*, and narrows rapidly to the midline, where the ornamented area is reduced to a point behind the distinct overlap area for the clavicle (od.Clav, Fig. 19A). There could not have been any midline contact between cleithra, in contrast to *Eusthenopteron* (m.ma, [3]: fig. 3E). There is no published illustration of the *Eusthenodon* cleithrum, but one specimen (P1481) was said to include “an almost complete cleithrum very suggestive of that in *Eusthenopteron*” ([19]: 68). By contrast, a previously unfigured cleithrum of *Mandageria* shows a similar pronounced anteromesial projection to *Edenopteron* (Fig. 19C), but it has a different, less triangular shape, with a rounded posteromesial angle (pa) reminiscent of *Eusthenopteron*. Incomplete elements representing the right shoulder girdle project off the collected slab of the holotype (Sh.g, Figs. 15, 16), and potentially more data could be obtained with further excavation.

#### Clavicle

The overlap for the clavicle is clearly seen (od.Clav, Fig. 19) because the right clavicle is preserved slightly twisted and displaced forward. There is no evidence of an ascending process on the clavicle, the relevant corner on the external impression being rounded and apparently complete. If present it must have been broken off, but nor is there any indication on the internal impression (preserved on piece d5). A new clavicle of *Eusthenodon waengsjoei* from Greenland ([36]: fig. 36) shows a prominent but completely unornamented ascending process. The external ornament is much coarser than on the holotype clavicle of *Edenopteron*, even though it is a slightly smaller example. *Edenopteron* possibly had an internal flange of the clavicle that fitted inside the bottom edge of the cleithrum (fl, Fig. 19A); the amount of removed bone with part and counterpart in position indicates a thickness of at least 12 mm for the ventral wall of the shoulder girdle. The clavicle is about 60 mm wide, with a gently convex posterior margin. Its anteromesial extremity is obscured under the right gular plate. The anterolateral margin is rounded, and slightly convex, where it would have fitted beneath the gular. The *Edenopteron* clavicle was evidently less elongate than in *Mandageria*, based on the specimen illustrated here (Fig. 19C).

#### Anocleithrum, Supracleithrum, Posttemporal, Extrascapulars

Only one of these bones can be described. A subrectangular element with indistinct overlap areas preserved just behind the left subopercular is interpreted as a supracleithrum, by comparison with *Mandageria*. A fracture across the middle with a sharp anterior inflection may represent the position of the sensory canal passage (llc, Fig. 18C), as preserved in *Mandageria* ([16]: fig. 10b). In that taxon these bones are differently developed to *Eusthenopteron* ([16]: 59), the supracleithrum being longer and four-sided, and the anocleithrum shorter and triangular (the reverse in *Eusthenopteron*). The anocleithrum and posttemporal of *Edenopteron* have not been identified, but can be restored after other tristichopterids (Fig. 23). The same applies to the extrascapulars, which would be expected in the holotype, given their life position immediately behind the post-parietal shield; as yet these have not been located in the prepared material. Based on the configuration of overlaps for the extrascapulars on the posterior skull margin (e.g. Figs. 11, 12) we assume the median extrascapular of *Edenopteron* had a broader anterior margin than the pointed shape of *Mandageria*.

### Postcranial Skeleton (Scales and Fins)

Because the right shoulder girdle of the holotype partly continues into the rock at the fossil site further excavation may reveal more elements from the postcranial skeleton. Some isolated scales and elongate elements interpreted as lepidotrichia are described here.

#### Fin skeleton

An assemblage of elements of various lengths (3–26 mm; 2–3 mm wide) on piece f3 (Fig. 20A) are interpreted as lepidotrichia probably from the pectoral fin, being located just caudal to the left cheek of ANU V3468. Most show a distinct central elongate ridge. The lepidotrichia of *Eusthenopteron* are generally simple, lacking ridges ([5]: fig. 18), but a few larger elements of the pectoral fin have been illustrated suggesting irregular ridges ([44]: fig. 7). Some ‘distal pectoral fin elements’ in *Sauripterus* likewise have suggestions of a longitudinal ridge ([45]: fig. 15), but the ridges illustrated here are more regular and distinct. A central ridge is also suggested on some lepidotrichia of the caudal and anal fins of *Mandageria* ([15]: fig. 18). The closest resemblance to the examples figured here are in pectoral fins from Canowindra for the tristichopterid *Cabonnichthys* ([16]: fig. 12C), and for the rhizodontid *Gooloogongia* ([10]: fig. 14b). Of interest is the fact that basal scutes on the fins, documented in Northern Hemisphere osteolepiforms and in *Eusthenopteron*, have not been found in the Canowindra tristichopterids [16].

Several other elongate elements, expanded at both ends like that illustrated from Grenfell, NSW ([17]: fig. 7), may be fin radials, for example partly exposed inside the right orbital notch of the holotype (?f.r, Figs. 9A, 10A), and on piece h4, an element 54 mm long and 9–10 mm diameter at both ends, narrowing to 4 mm diameter in the middle region.

#### Scales

Isolated scales are scattered through the matrix surrounding the dermal bones of *Edenopteron*, mostly exposing the internal surface that shows clearly the drop-shaped central boss characteristic of tristichopterids and rhizodontids (Fig. 20C, D). The boss is situated more towards the anterior edge as in *Eusthenopteron* and *Eusthenodon* ([19]: fig. 30). One small scale (8 mm across; part and counterpart on piece h3), possibly from ANU V3468, has a notched margin where crossed by an oblique internal ridge, probably a sensory canal (llc, Fig. 20D). The external surface preserved as an impression is indicated by a few examples where portion of the scale has broken away (Fig. 20B, C). These suggest widely spaced grooves (gr), as in the distinctive ‘mandageriid’ scale morphology of the Canowindra tristichopterids previously described [20]. Another incomplete scale (on piece h2) seems to lack the grooves. We cannot attribute these isolated scales to particular specimens, so it is unclear whether this was a variable feature of *Edenopteron*. The grooves are much more closely spaced in *Langlieria* scales from Belgium ([37]: fig. 13) but these do have a relatively large boss, as in Figure 12D. In scales of *Hyneria* the boss is relatively smaller [33], and an indented posterior edge is characteristic of this taxon [41]. This grooved scale surface is distinctly different from the scales of Northern Hemisphere *Eusthenodon* [20], as exemplified by the Greenland type species ([19]: fig. 30), and also demonstrated in *Jarvikina wenjukovi* from Russia ([40]: pl. 8, fig. 1a). Based on that taxonomic treatment, such scale ornament is typical of several Northern Hemisphere genera, in the same way that the grooved scales typify at least four Southern Hemisphere genera: *Mandageria* and *Cabonnichthys* from Canowindra, *Edenopteron* as documented here, and probably also *Notorhizodon* from Antarctica ([20]: fig. 4C). This scale type has also been documented from a third Australian locality, Jemalong NSW ([9]: fig. 9B).

### Tooth Histology

Tooth structure is not well preserved in the material. For the holotype, palatal fangs are mainly preserved as impressions, while the dentary and first coronoid fangs of the left lower jaw are intact and partly exposed (Fig. 17B, C). The more posterior fangs are embedded in matrix. An isolated incomplete tooth or fang shows an open pulp cavity about half way along its length (Fig. 20E). The weathered base of the left vomerine fang of ANU V3468 (Fig. 20F) shows distinct extensions of the pulp cavity separating foldings in the dentine (plicidentine). As with the fangs of the dermopalatine and ectopterygoid (Fig. 4B) the base and lower part are rounded in section. Vomerine fangs are generally of rounded shape (P. Ahlberg, pers. comm. 4 July 2012), whereas others become flattened with cutting edges towards the tip (e.g. *Eusthenodon waengsjoei* ([16]: fig. 8B). The cusp of *Edenopteron* fangs is only exposed on the left first coronoid of the holotype; it is strongly laterally compressed, with cutting edges (7 mm across anteroposteriorly; only 3 mm labiolingually, measured ∼13 mm from the tip). This is more strongly compressed than an illustrated coronoid fang of *Langlieria* ([37]: fig. 12B).

Some histology of the vomerine fang is indicated in CT scans of the holotype (Fig. 21). Detailed histology will be described elsewhere, and here we give a brief comparison with the ‘eusthenodont’ type of Schultze [46], [47]. This type, based on *Eusthenodon*, but also identified in *Platycephalichthys* and *Litoptychus*, was defined by three features [47]: i) pulp cavity filled with osteodentine; ii) folding of orthodentine often more complicated than ‘polyplocodont’ type; iii) bone of attachment extending between folds. In the *Edenopteron* fang the basal third (Fig. 21C–D) shows a pulp cavity either open or filled with matrix (pc), and possibly bone of attachment (b.a) enters the plications, although this is unclear. The next higher section shows a narrower cavity apparently encircled by osteodentine, and this tissue completely fills the pulp cavity closer to the fang tip (ost, Fig. 21A–B). This open lower pulp cavity corresponds to the coronoid fang reconstruction for *Eusthenodon* ([46]: fig. 2). In all four sections of the *Edenopteron* fang the surrounding folded orthodentine (plicidentine) shows a distinctive pattern of bifurcating extensions outward from the pulp cavity, at least 18 near the base, reducing to 13 in the uppermost section. This is rather different from previous illustrations of fang sections for the Northern Hemisphere genera *Platycephalichthys* ([34]: fig. 21; [40]: figs. 25, 27) and *Eusthenodon* ([46]: pls. 17–19), the only similar previous illustration being a partial section through the base of a coronoid fang of *Eusthenodon waengsjoei* from Greenland ([46]: pl. 17, fig. 5a).

For Southern Hemisphere taxa, tooth histology is unknown in the Canowindra tristichopterids, *Notorhizodon*, and also *Marsdenichthys*, all of which were negatively prepared from impressions. It is surprising that the only published illustrations of sarcopterygian tooth histology from the Upper Devonian of southeastern Australia include a fang with very similar bifurcating ‘channels’ ([48]: fig. 3f). This fang is at least 6 cm long, and comes from the Genoa River Beds, a locality in eastern Victoria ∼60 km south-west of the Boyds Tower locality (Fig. 1). This fang was assigned to Porolepiformes because there was no bone of attachment between the plications, but since the base of the tooth and surrounding bone was not preserved this seems unwarranted, and an associated large but very incomplete lower jaw may belong to a tristichopterid [48]. In contrast, the histology of an isolated tooth of *Hyneria* from the Upper Devonian of the eastern United States [49] is completely different to that shown here, and also shows little resemblance to *Eusthenodon*. A sectioned tristichopterid coronoid fang from the Upper Devonian of Belgium ([37]: fig. 12) shows typical eusthenodont histology.

## Discussion

### Relationships of *Edenopteron*

This new taxon is provisionally included in the tristichopterid subfamily Mandageriinae on the evidence summarised above. The detailed description of *Edenopteron* provides numerous additional characters to be assessed in other tristichopterid taxa for a more robust phylogenetic analysis (beyond the scope of the present paper). Previously, tristichopterid phylogeny was analysed with a character matrix of only 14 characters for seven taxa [16], recently updated to 22 characters for nine taxa [30]. A recent analysis [50] uses a much larger data set that includes both dipnomorphs and tetrapods. This effectively dilutes characters relevant to tristichopterid monophyly, with the result that some genera (e.g. *Platycephalichthys*) fall outside the group. *Notorhizodon* is a key Gondwanan taxon not included in that analysis, and the ‘mandageriid’ characters discussed above [20] were also overlooked. The description of *Edenopteron* adds to uncertainty about some characters previously used to ally Australian taxa to Northern Hemisphere forms. For example, *Edenopteron* shows the separation of the intertemporal from the posterior supraorbital, previously used as a shared character linking *Mandageria* from Canowindra to *Eusthenodon* from Greenland, and excluding the second Canowindra tristichopterid *Cabonnichthys* [15], [16]. On the other hand the two Canowindra taxa resemble each other, and *Eusthenodon*, but differ from *Edenopteron*, in the shape of the lachrymal with a more prominent preorbital portion. Some key characters observed only in Australian forms, like the accessory vomers (identified above in *Edenopteron*) were not taken into account in the first analysis of the Canowindra tristichopterids. Later [30], this character has been scored ‘?’ for *Eusthenodon*, presumably on the evidence of the Australian ‘*Eusthenodon*’ reported to have accessory vomers [15], [21]. This is the material mentioned in the Introduction from a second site about 10 km down the coast from the *Edenopteron* type locality. Field latex casts of one collected skull (AMF 134129) and an associated palate provided the features distinguishing that form from *Eusthenodon* ([20]: 330). The palate belonging to this skull awaits excavation, but the casts clearly show both accessory vomers. In contrast, well preserved palates of the Greenland *Eusthenodon* with surrounding bones in place ([19]: pl. 16) clearly demonstrate that accessory vomers were absent. AMF 134129 shows also that the jugal reached the orbit, as in *Edenopteron* but unlike *Eusthenodon*, so it could be closely related to, or the same as our new taxon. What is clear from currently available evidence is that *Eusthenodon* sensu stricto (from the East Greenland type locality), and in fact all other Northern Hemisphere tristichopterids, should be scored as lacking accessory vomers. Similarly, the remains from Grenfell, NSW, assigned to *Eusthenodon* [17], [18] should be treated with caution until more complete remains are found, given that the above description of *Edenopteron* shows numerous differences in morphology, even if there are close similarities in certain dermal bones.

### Size of *Edenopteron*

The type material of *Eusthenodon* from the East Greenland Famennian includes specimens indicating a skull length approaching 50 cm ([19]: 58), giving an estimated body length of 2.25–2.5 m (using assumed proportions of body length 4.5–5 times skull length). This was based on estimations from incomplete skull portions; e.g. a parietal shield 220 mm long, a post-parietal shield 84 mm long ([19]: fig. 24), and a cheek about 36 cm long. The lower jaw of *Eusthenodon* is proportionately longer than in other forms ([19]: 55, 64), being almost four times longer than post-parietal shield length. The reconstruction of *Edenopteron* suggests more elongate jaws than this, approaching 4.5 times post-parietal length. Lower jaw length is 5.7 times the depth in the *Eusthenodon* reconstruction [19]. Documented from the Belgian Famennian is a large jaw of *Langlieria* about 37 cm long and 60 mm high, indicating a fish with a skull roof (excluding extrascapulars) about 300 mm long, comparable to the largest *Eusthenodon* from Greenland, and also the largest *Platycephalichthys* and *Hyneria* [37]. These comparisons were based on a survey of lower jaw length compared to skull length in various osteolepiforms, indicating that they showed fairly constant proportions [51]. The reconstruction of the *Hyneria* skull inferred a much longer parietal than post-parietal shield, but because the parietals were unknown this also relied on assumed constant proportions to jaw length, even though the lower jaw of the *Hyneria* holotype as figured is incompletely exposed ([33]: fig. 4). There is much new material of *Hyneria* [41], and the largest complete jaw found so far (ANSP 21432) is 38 cm long; a much larger fragment of the dentary and jaw symphysis (ANSP 21434) could have come from a jaw approaching twice that length (T. Daeschler, pers. comm., 3 March 2009). However there are also rhizodontids in the Red Hill fauna [52], this group also having dentary fangs in the jaws [53]. From Russia are known *Eusthenodon* skulls up to a maximum of 40 cm long, and *Platycephalichthys* jaws 30–45 cm long (O. Lebedev, pers. comm., 5 March 2009). The lower jaw of *Notorhizodon* was restored at about 40 cm long, indicating a total length for the fish of 3+ m based on *Screbinodus* proportions, or 2.6 m based on *Eusthenopteron* ([8]: 35). Published reconstructions for the Canowindra tristichopterids indicate total lengths of about 6.1 times jaw length for *Mandageria* [15], and about 6.6 times jaw length for *Cabonnichthys* [16].

The lower jaw of the *Edenopteron* holotype is about 48 cm long. Based on the above comparisons, a total length in the range 2.9–3.2 m can be estimated, making this specimen probably the largest Devonian tristichopterid found so far that is known from associated semi-articulated remains. Our reconstruction of the head (Figs. 22, 23A–C) indicates a skull length (excluding extrascapulars) of about 30 cm (length of parietal shield 18.8 cm, and post-parietal shield 11.1 cm). On the other hand, incomplete remains from other localities, like isolated jaw fragments, indicate that much larger sarcopterygians existed. Carboniferous rhizodontids were the largest sarcopterygians (and perhaps the largest known non-marine osteichthyans), reaching 6–7 m long [45], but even Devonian Sarcopterygii may have approached this size. An isolated maxilla of *Onychodus* sp. (Delaware Limestone, USA) is nearly 30 cm long, suggesting a total length for the fish of about 4 m (J.A. Long, pers. comm., 19 Sept. 2012). Other examples include a lower jaw fragment from Russia (possibly the porolepiform *Holoptychius*) 7–10 cm deep, and numerous *Holoptychius* scales 8–10 cm in diameter (O. Lebedev, pers. comm., 5 March 2009).

### Biostratigraphy and Biogeography

The age evidence for the Worange Point Formation fish assemblage that includes *Edenopteron* gen. nov. includes palynology (but from other localities) indicating a late Famennian age. There is also a smooth species of the placoderm *Groenlandaspis*, suggesting a similar age to the Grenfell assemblage of central NSW [22], from which bones assigned to *Eusthenodon* have been recorded (see above). However, there are differences in shape of the *Groenlandaspis* bones from the two localities ([9]: fig. 4), and the distinctive porolepiform scales from Grenfell [17] have never been found in the Worange Point Formation, nor the sinolepid antiarch *Grenfellaspis*, so an age difference within the Famennian seems likely.

Evidence of an East Gondwanan mandageriid subgroup of tristichopterids [20] represents broad biogeographic congruence with the East Gondwanan distribution of the family Canowindridae as previously documented [8]. Considering the biogeography and relationships of both tristichopterids and canowindrids, the validity of the latter group depends on the (reconstructed) basal scutes of the pectoral fin in *Koharalepis* ([54]: 221–23). The recognition of an East Gondwanan tristichopterid subgroup (Mandageriinae) contradicts previous biogeographic arguments that more primitive tristichopterids originated in the Northern Hemisphere (Laurussian continent), and that more derived tristichopterids were a “widely distributed and freely dispersing fauna” perhaps originating in Gondwana [16]. Such arguments rely on both Hennig’s and Matthew’s ‘rules’ of biogeography (respectively, that a center of origin is indicated either by the occurrence of basal clades, or by the oldest fossil representatives). The supporting biogeographic pattern has been termed an H–M pattern [55].

On the reinterpretation that *Notorhizodon* was a derived tristichopterid [10], it was later suggested [18] that the latter group (and all tristichopterids) originated in Laurussia, and that derived tristichopterids expanded into Gondwana as early as Givetian (on the evidence of *Notorhizodon*). However the supporting phylogenetic analysis showed the Antarctic form and the Russian genus *Platycephalichthys* in an unresolved trichotomy with other ‘derived’ tristichopterids, and *Platycephalichthys* is supposedly younger (mid-Frasnian [21]). The first tristichopterid data matrix was run with three different outgroups, as the resulting phylogeny was very sensitive to outgroup choice [16]. The preferred result was favoured on three grounds: it was completely resolved, it provided a “perfect stratigraphic fit”, and was consistent with a “gradually lengthening anterior cranial division in the Tristichopteridae”. On the third point, it is noted that *Edenopteron* gen. nov., although late Famennian in age, has a less elongate parietal shield than the older (Frasnian) *Mandageria* and *Cabonnichthys*. Regarding stratigraphic fit, we note that the late Middle Devonian of Nevada has produced both a tristichopterid with ‘derived’ features [31], and a similar taxon (*Tinarau* Swartz, 2012 [49]), placed (with *Platycephalichthys* from Russia) outside the tristichopterid clade (but some specimens assigned to this taxon are considered to belong to the tristichopterid *Bruehnopteron* [31]). Applying the biogeographic ‘rules’ of Hennig (origin indicated by the most primitive clades) and Matthew (origin indicated by the oldest fossils) to the phylogeny of Swartz [49] would resolve a South China–Gondwana origin for all tetrapodomorphs, because the Early Devonian *Kenichthys* is the outgroup, and Rhizodontidae are the next crownward clade, with the oldest known rhizodontids in Antarctica [8], [10] and possibly earlier in central Australia [56]. Crownward of rhizodontids are the Gondwana taxa *Marsdenichthys* and Canowindridae. On the other hand, plotting only two areas (Laurussia or Gondwana) onto the cladograms of Johanson [18] gives a biogeographically inconclusive result (an ‘I-pattern’ [55]). Given uncertainties of dating and incompleteness of the fossil record, current evidence is only sufficient to indicate that tristichopterid-like tetrapodomorph fishes, including both presumed ‘primitive’ and ‘derived’ forms, appeared at about the same time in both hemispheres.

It is now widely accepted that a major faunal interchange between southern and northern paleocontinents occurred during the Middle–Late Devonian, within which many fish groups show complex distribution patterns in time and space suggesting either Asian, Gondwanan or Laurussian evolutionary origins, followed by later dispersal [57], [58]. How the geographic origins of tetrapodomorphs generally, or some significant subgroups (e.g. tristichopterids, tetrapods) relate to these patterns remains very uncertain due to sparse data from Gondwana, the least researched but largest landmass of the Devonian. Further resolving biogeographic hypotheses with regard to tristichopterids will rely heavily on deciding the outgroup for tristichopterids. As noted above, *Marsdenichthys* from Victoria was first suggested [6] to be the sister group to all other tristichopterids because of a unique character combination – an ‘extratemporal’ bone in the skull rather than a ‘postspiracular’, plus round non-cosmoid scales with an inner boss (morphology recently confirmed [7]). An alternative phylogenetic analysis [28] has placed the Northern Hemisphere (Greenland) taxon *Spodichthys* as the sister group to other tristichopterids (it has the same character combination as *Marsdenichthys*), but the Southern Hemisphere *Marsdenichthys* was not included in that data matrix. A new comprehensive phylogenetic analysis, including new characters resulting from the above descriptions, scored for all taxa from both hemispheres relevant to the question of tristichopterid origins and relationships, must be undertaken before biogeographic implications can be taken any further.

## Supporting Information

Information S1 **Specimen curation and storage list for** ***Edenopteron keithcrooki*** **gen. et sp. nov., and associated material.**
(PDF)
