# Supplementary material for: A Gigantic Sarcopterygian (Tetrapodomorph Lobe-Finned Fish) from the Upper Devonian of Gondwana (Eden, New South Wales, Australia)
Source: PLoS One. 2013 Mar 6;8(3):e53871. doi: 10.1371/journal.pone.0053871 (PMC3590215; doi:10.1371/journal.pone.0053871)
Supplement: Information S1 — Specimen curation and storage list for Edenopteron keithcrooki gen. et sp. nov., and associated material. (PDF) [file pone.0053871.s001.pdf]

Edenopteron material: Curation and Storage						
[note: for ANU Fossil database add prefix 7; i.e V3479 = ANU 73479]						
Drawer Number	Block Layer	Block Number	ANU Reg. No.	Determination	Assoc. material	Determination
1	a	1	V3479	Clavicle&Cleithrum		
1	a	2	V3479	Clavicle&Cleithrum		
1	a	3	V3479	Fragment Jaw		
2	a	4	V3426	Parietal Outer	V3479	Cleithrum Outer
1	a	5	V3479	Frag. Jaw		
1	a	6	V3479	Jaw		
1	a	7	V3426	Snout Outer		
1	a	8	V3426	Snout Inner		
1	a	9	V3426	Vomer Left		
7	a	10	V3426	Maxilla	V2378	*Remigolepis* Head
2	a	11	V3426	Post Parietal Outer		
2	a	12	V3426	Cheek Left Outer		
2	a	13	V3426	Snout Inner		
2	a	14	V3426	Snout Outer		
3	a	15	V3426	Parietal Outer		
3	b	1	V3426	Cheek Right	V3479	Cleithrum
2	b	2	V3426	Maxilla		
2	b	3	V3426	Vomer Right		
3	b	4	V3426	Parietal Outer		
3	b	5	V3426	Palate & P. Parietal Inner		
3	b	6	V3426	Gular & Cheek Left		
1	b	7	V3426	Palate & Lower Jaws		
4	b	8	V3426	Mx, Cheek Rt & Lower Jaws		
3	b	9	V3426	Palate & Lower Jaws		
3	b	10	V3426	Cleithrum		
4	c	1	V3426	Jaw articulation		
2	c	2	V3426	Maxilla		
3	c	3	V3426	Parietal Outer		
6	c	4	V3426	Cheek & Jaw Left Outer		
3	c	5	V3426	Jaw Lower		
5	c	6	V3426	Clm Inside		
5	c	7	V3426	Sop inside&Clm dorsal edge		
5	c	8	V3426	Operculum Ext		
5	c	9	V3426	Gular Internal		
4	d	1	V3426	Jaw Lower		
6	d	2	V3426	Cleithrum & Clavicle Outer		
3	d	4	V3426	Rt. Cheek Int., Mx		
6	d	5	V3426	Clavicle Outer		
3	d	6	V3426	Post Parietal Outer		
4	d	7	V3426	Add. Fossa & Cheek Rt.		
6	d	8	V3426	Add Fossa		
6	d	9	V3426	Jaw Lower & Cleithrum Outer		
11	d	10	V3471	*Remigolepis* Head		

7	e	1	V2378	*Remigolepis* Head		
7	e	2	V2378	*Remigolepis* Body [ext.]		
7	e	3	V2378	*Remigolepis* Tail		
7	e	4	V2378	*Remigolepis* Tail		
7	e	5	V2378	*Remigolepis* Head+Body [int.]		
11	e	6	V3471	*Remigolepis* Lateral		
11	e	7	V3471	*Remigolepis* Ventral		
7	e	8	V2378	*Remigolepis* Head		
7	e	9	V2378	*Remigolepis* Head		
13	e	10	V3471	*Remigolepis* Head		
8	f	1	V3478	indeterminate		
8	f	2	V3478	Skull, cheek [c/part to f4]		
8	f	3	V3478	fin ray		
8	f	4	V3478	Skull, cheek, fin ray [c/part to f2,f3]		
10	g	1	V3478	indet. Bones	V3469	*Remigolepis* ventral
10	g	2	V3478	?Pmx tusks [c/part to h2]		
10	g	3	V3478	?Cheek [inner]		
10	g	4	V3468	Lower Jaw [inner]		
9	g	5	V3468	Cheek [inner]		
12	g	6	V2378	*Remigolepis* Head		
12	g	7	V3470	*Remigolepis* Ventral		
12	g	8	V3470	*Remigolepis* Lateral		
10	g	9	V3468	Adductor fossa		
13	g	10	V3471	*Remigolepis* Lateral		
12	g	11	V3470	*Remigolepis* PMD		
10	g	12	V3478	?Pmx tusks [c/part to h2]		
10	h	1	V3478	skull portion		
10	h	2	V3478	tusk pair, fin ray, scale [c/part to g2]		
9	h	3		scale [p+c/part]		
13	h	4		fin element		
14	h	5		isolated tooth [fig.]		
14	h	6		isolated tooth [tip intact]		
14	h	7		isolated tooth [p+c/part]		
9	h	8	V3468	Cheek, lower jaw [oute	V3470	*Remigolepis* Ventral
15			V3468	unassigned fragments		
15			?	unassigned fragments		
16			V3474	holoptychiid scales		
16			V3475	holoptychiid scales		
16			V3476	smooth bones [?lungfish]		
16			V3477	small osteichthyan jaw		
16			?	unassigned fragments		
